# New cytotoxic dammarane type saponins from *Ziziphus spina-christi*

**DOI:** 10.1038/s41598-023-46841-2

**Published:** 2023-11-23

**Authors:** Abeer H. Elmaidomy, Amr El Zawily, Aliasger K. Salem, Faisal H. Altemani, Naseh A. Algehainy, Abdullah H. Altemani, Mostafa E. Rateb, Usama Ramadan Abdelmohsen, Nourhan Hisham Shady

**Affiliations:** 1 Department of Pharmacognosy, Faculty of Pharmacy, Beni-Suef University, Beni-Suef, 62511 Egypt; 2 Department of Plant and Microbiology, Faculty of Science, Damanhour University, Damanhour, 22511 Egypt; 3 Division of Pharmaceutics and Translational Therapeutics, College of Pharmacy, University of Iowa, Iowa City, IA 52242 USA; 4 Department of Medical Laboratory Technology, Faculty of Applied Medical Sciences, University of Tabuk, 71491 Tabuk, Saudi Arabia; 5 Department of Family and Community Medicine, Faculty of Medicine, University of Tabuk, 71491 Tabuk, Saudi Arabia; 6 School of Computing, Engineering & Physical Sciences, University of the West of Scotland, Paisley, PA1 2BE UK; 7 Department of Pharmacognosy, Faculty of Pharmacy, Minia University, Minia, 61519 Egypt; 8 Department of Pharmacognosy, Faculty of Pharmacy, Deraya University, Universities Zone, New Minia, 61111 Egypt

**Keywords:** Cancer therapy, Drug discovery, Plant sciences, Chemistry

## Abstract

Cancer is the world's second-leading cause of death. Drug development efforts frequently focus on medicinal plants since they are a valuable source of anticancer medications. A phytochemical investigation of the edible *Ziziphus spina-christi* (F. Rhamnaceae) leaf extract afforded two new dammarane type saponins identified as christinin E and F (**1, 2**), along with the known compound christinin A (**3**). Different cancer cell lines, such as lung cancer (A549), glioblastoma (U87), breast cancer (MDA-MB-231), and colorectal carcinoma (CT-26) cell lines, were used to investigate the extracted compounds' cytotoxic properties. Our findings showed significant effects on all the tested cell lines at varying concentrations (1, 5, 10, and 20 µg/mL). The three compounds exhibited potent activity at low concentrations (< 10 μg/mL), as evidenced by their low IC_50_ values. To further investigate the complex relationships between these identified cancer-relevant biological targets and to identify critical targets in the pathogenesis of the disease, we turned to network pharmacology and in silico-based investigations. Following this, in silico-based analysis (e.g., inverse docking, ΔG calculation, and molecular dynamics simulation) was performed on the structures of the isolated compounds to identify additional potential targets for these compounds and their likely interactions with various signalling pathways relevant to this disease. Based on our findings, *Z. spina-christi's* compounds showed promise as potential anti-cancer therapeutic leads in the future.

## Introduction

Uncontrolled cell proliferation characterises a category of illnesses known as cancer^1^. This unchecked cell development has the capacity to infect local and far-off tissues, potentially leading to potentially fatal problems^2^. Cancer is a worldwide public health issue and one of the main causes of death in both developing and industrialised countries^2^. According to a World Health Organisation (WHO) epidemiological analysis, cancer killed 7.6 million people in 2018, with the number anticipated to quadruple by 2030^2^. To cure cancer, several treatment approaches have been explored, the most prevalent of which being chemotherapy. This treatment entails the use of medications or chemical agents to eliminate fast dividing cells, thereby preventing their spread to other normal bodily cells. Despite chemotherapy's success rate, patients_-_continue to experience a variety of side effects, including overall weakness, exhaustion, lack of appetite, and infections. Furthermore, the FDA-approved anticancer drugs' lack of selectivity and toxicity have resulted in a significant disadvantage in cancer treatment^3^. As a result, the search for novel drugs for treating cancer is crucial.

Phytochemical compounds, which are essentially secondary metabolites that plants need to sustain their survival and fecundity, are found in traditional medicinal plants^4–6^. A range of secondary metabolites, including as glucosinolates, alkaloids, triterpenoids, flavonoids, saponins, pigments, and tannins, are medicinally important phytochemical substances^7,8^. Numerous investigations on the use of secondary metabolites of plants in conventional medicine have been done^9–11^. These secondary metabolites were found to have anti-inflammatory, antimicrobial, antiviral, cardioprotective, and anticancer effects^12,13^. Approximately 60% of anticancer medicines are in clinical trials or preclinical testing e.g. vincristine, vinblastine, paclitaxel, bleomycin, camptothecin, etoposide, curcumin, irinotecan, genistein, resveratrol, topotecan, lycopene, allicin, diosgenin, dactinomycin, *β*-carotene, doxorubicin, and camptothecin are derived from plants^14–16^.

Plant-derived medications are sought after for anticancer treatment since they are both natural and widely available. They are easily supplied orally as part of the patient's diet^17,18^. Furthermore, because they are naturally generated from plants, they are often more tolerated and non-toxic to normal human cells^19^. However, there are exceptions such as lectins, lignans, cyanogenetic glycosides, saponins, and some taxanes^19^. If plant-derived medications are non-toxic to normal cell lines with selective cytotoxicity against cancer cell lines, they can be advanced to clinical trials for further therapeutic development. Plant-derived medications can be classified into four types based on their activities: methytransferase inhibitors, DNA damage preventive agents or antioxidants, histone deacetylases (HDAC) inhibitors, and mitotic disruptors^18^. HDAC inhibitors include compounds such as sulforaphane, isothiocyanates, isoflavones, and pomiferin. They stop carcinogenic proteins from working. Sulforaphane, for example, has been demonstrated to block key targets in breast cancer development. HDAC inhibition by sulforaphane resulted in decreased expression of ER, EGFR, and HER-2 in breast cancer cell lines^20^. HDAC inhibitors reactivate epigenetically suppressed genes that are functional for chromatin acetylation in cancer cells, allowing cancer cells to apoptose. Plant-derived chemicals that suppress HDAC may improve chemotherapeutic sensitivity in human malignancies^18,20^. Vinca alkaloids derivatives, vincristine, vinblastine, vinorelbine, vindesine, and vinflunine are medicines that suppress microtubule dynamics by binding to β-tubulin. Microtubule disruptors include taxanes such as paclitaxel and its counterpart docetaxel. These substances prevent cell cycle phase transitions from metaphase to anaphase, resulting in cell cycle arrest and apoptosis^21,22^. Paclitaxel inhibits cancer cell replication by stabilising or polymerizing microtubules in the cells^21–25^. Paclitaxel was one of the first medications to have a significant influence on cancer treatment, while vincristine and vinblastine were two of the first drugs identified^25^.

Combinations of medications generated from plant extracts of vinca alkaloids, Taxus diterpenes, Podiphyllum lignans, and Camptotheca alkaloids may boost their anticancer effects and efficacy as therapeutic agents^25,26^. Extracts from *Urtica membranacea, Artemesia monosperma*, and *Origanum dayi*^26^ were tested against cancer cell lines (lung, breast, colon, and prostate malignancies). The study found that plant extracts containing a combination of anticancer chemicals were capable of killing cancer cells while having no effect on normal human lymphocytes and fibroblasts. This makes plant extracts more appealing as therapeutic agents than chemically generated medicines, which create hazardous problems in cancer treatment. Plant extracts caused apoptosis, as evidenced by an increased sub-G1 phase population of cells with reduced DNA content and chromatin condensation. After extract administration, there was also an increase in caspase 3 activation, which is a crucial stage in apoptotic cell death^26^. Clinical trials may be explored for anticancer cancer medicines that have selectivity for cancer cells, may trigger cell death, and prevent tumour growth.

Research interest in additional secondary metabolites, like saponins, has increased as a result of the notable success so far in employing natural substances as chemotherapeutic alternatives^27^. Saponins are a structurally varied class of phytochemicals found in higher plants, marine creatures, and microbes. This group exhibited a variety of pharmacological activities, including antiviral, anti-inflammatory, immunoregulatory, cardioprotective, and anticancer actions^28,29^. The strong effect that saponins have on cancer cells has drawn a lot of attention from researchers in the pharmaceutical industry. These substances have shown exceptional promise in both in vitro and in vivo settings in inhibiting various cancer cells. Despite the significant advancements made recently, the toxicity and poor pharmacokinetic features of saponins as an anticancer drug have presented some challenges^27^.

Wild *Z. spina-christi* trees are well known as "Christ's thorn jujube." *Z. spina-christi* is one of the most important food sources since it has a variety of nutritious components^9^. The plant contains a variety of bioactive phytochemicals, with cyclopeptide, alkaloids (spinanine A), tannins, sterols, peptide, flavonoids (rutin and quercetin), saponins (christanin A-D, and betulinic acid), triterpenoids, sapogenins, and triterpenic acids making up the majority of these compounds^30–35^. *Z. spinachristi* was observed to produce antibacterial, antifungal, antinociceptive, antioxidant, anti-inflammatory action, antiallergic, and anti-influenza^36–41^. Previous research on the beneficial activities of *Z. spina-christi* related to the nervous system of being anxiolytic, analgesic, and anti-depressant have been also determined^42–44^.

In the present study, three compounds were isolated and identified from the leaf methanol extract of *Z. spina-christi*. Among them, two new dammarane-type saponins christanin E–F (**1**–**2**), were obtained. All isolated compounds were evaluated for their cytotoxic activities against human lung cancer (A549), human glioblastoma (U87), human breast cancer (MDA-MB-231), and colorectal carcinoma (CT-26) cell lines. Additionally, the isolated compounds underwent molecular docking to determine the possible mechanisms responsible for this activity.

## Materials and methods

### Plant collection

*Z. spina-christi* leaves were collected in May 2022 from Minia Governorate, Egypt, where the permissions were obtained from an appropriate governing body to a piece of legislation that permits this. The plant was kindly identified and authenticated by Abdallah Salem, Minia, Egypt comply with the IUCN Policy Statement on Research Involving Species at Risk of Extinction and the Convention on the Trade in Endangered Species of Wild Fauna and Flora. A voucher specimen (2021-BuPD 111) was deposited at Beni-Suef University's Department of Pharmacognosy, Faculty of Pharmacy.

### Chemicals and reagents

El-Nasr Company for_-_Pharmaceuticals and^-^Chemicals (Egypt) supplied the solvents utilised in this study, which comprised n-hexane (n-hex.), ethyl acetate (EtOAC), dichloromethane (DCM), n-butanol (n-but.), and methanol (MeOH), ethanol. Sigma-Aldrich (Saint Louis, Missouri, USA) provided the deuterated solvents used in the chromatographic and spectroscopic investigations, including dimethyl sulfoxide-d6 (DMSO-d6). Polyamide-6 (50–160 m), silica gel 60 (63–200 m, E. Merck, Sigma-Aldrich), and sephadex LH20 (0.25–0.1 mm, GE Healthcare, Sigma-Aldrich) were used for column chromatography (CC). Vacuum liquid chromatography (VLC) was done using silica gel GF254 (El-Nasr_-_Company for_-_Pharmaceuticals and Chemicals,^-^Egypt). Pre-coated silica gel 60 GF254 plates_-_(E. Merck, Darmstadt,^-^Germany; 20 × 20 cm, 0.25 mm in_-_thickness) were used for the thin-layer_-_chromatography^-^(TLC) procedure. Spraying^-^the spots with the_-_para-anisaldehyde_-_(PAA) reagent (85: 5: 10: 0.5 absolute_-_EtOH: sulfuric^-^acid: glacial_-_acetic acid: para-anisaldehyde), the specks were visible after heating them to 110 °C^45^.

### Spectral analyses

At 400 and 100 MHz, respectively, proton ^1^H and ^13^C Distortionless Enhancement by Polarisation Transfer-Q (DEPT-Q) NMR spectra were captured. In dimethyl sulfoxide-d6 (DMSO-d6), tetramethylsilane (TMS) was utilised as an internal standard, with the residual solvent peak (*δ*_H_ = 2.50 and *δ*_C_ = 39.5) serving as references, respectively. The Bruker Advance III 400 MHz with BBFO Smart Probe and the Bruker 400 MHz AEON Nitrogen-Free Magnet were used for the measurements (Bruker AG, Billerica, MA, USA). Carbon multiplicities were determined using a DEPT-Q experiment. The UV spectrum of methanol was measured using a Shimadzu^-^UV 2401PC_-_spectrophotometer (Shimadzu^-^Corporation,_-_UV-2401PC/UV-2501PC,^-^Kyoto,^-^Japan). The infrared (IR)_-_spectra were measured using a Jasco_-_FTIR 300E_-_infrared spectrophotometer. HRESIMS_-_data were collected using an Acquity^-^Ultra Performance^-^Liquid Chromatography^-^system linked to a Synapt^-^G2 HDMS_-_quadrupole time-of-flight hybrid mass^-^spectrometer (Waters,^-^Milford, MA,^-^USA).

### Extraction and fractionation of* Ziziphus spina-christi*

Fresh *Z. spina-christi leaves* (1 kg) were harvested and air-dried^-^for one week in the shade. The finely powdered leaves were macerated in 70% methanol (5 L, 3 days each) at room temperature, and the crude extract was concentrated using a rotary^-^evaporator (Buchi Rotavapor_-_R-300, Cole-Parmer,^-^Vernon Hills,^-^IL, USA) under vacuum at 45 °C. The dried extract was suspended in 500 mL of distilled water (H_2_O), then portioned with various polarity solvents (*n*-Hex.,^-^DCM, EtOAC,_-_and *n*-but.). In each step, the organic phase was evaporated at decreased pressure to obtain fractions I (10.0 g), II (5.0 g), III (12.0 g), and IV (20.0 g), while the^-^remaining mother_-_liquor was concentrated to yield the^-^aqueous fraction (V). For biological and phytochemical research, all fractions were kept at 4 °C^46–52^.

### Isolation and purification of compounds

Fraction IV (5 g) was fractionated on a polyamide-6 column (50–160 μm, 1000 × 5 cm, 100 g) using gradient elution starting with water (H_2_O) and ending with MeOH in the order of increasing polarities (0, 5, 10, 15, 20, 25, 30, 35, 40, 45, 50, 60, 80 and 100%, 1000 mL each, FR 5 mL/min). The effluents from the column were collected in fractions (250 mL each); and each collected fraction was concentrated and monitored by TLC using the system EtOAc*:* acetic acid: formic acid: H_2_O 10: 1: 1: 2 and detected using PAA reagents. Similar fractions were grouped and concentrated under reduced pressure to provide two sub-fractions (I_1_–I_3_). Subfraction I_1_–I_3_ (1.0 g, each) were further purified separately on sephadex LH_20_ column (0.25–0.1 mm, 100 × 0.5 cm, 100 gm) which was eluted with MeOH to afford compound **1** (37 mg), **2** (10 mg), and **3** (18 mg).

Christinin E (1): white powder; [UV (MeOH) *λ*_max_ (log_ε_) 280 (5.5), 270 (6.0), 300 (4.5) nm; IR υ_max_ (KBr) 3600, 3500, 3400, 3100, 1730, 1350, 1300, 1000 cm^−1^; NMR data; see Tables 1 and 2; HRESIMS m/z 1059.5559 [M + H]^+^ (calc. for C_56_H_83_O_19_, 1059.5529).

**Table 1:** DEPT-Q (400 MHz) and ^1^H (100 MHz) NMR data of compounds **1, 2, 3** in DMSO-*d*_*6*_; Carbon multiplicities were determined by the DEPT-Q experiments.

Nu	1		2		3	
	^*δ*^_C_	^*δ*^_H_ (*J* in Hz)	^*δ*^_C_	^*δ*^_H_ (*J* in Hz)	^*δ*^_C_	^*δ*^_H_ (*J* in Hz)
1	38.6, CH_2_	0.87, 1.58, *m*	38.5, CH_2_	0.87, 1.58, *m*	38.6, CH_2_	0.87, 1.58, *m*
2	26.2, CH_2_	1.52, 1.70,* m*	26.2, CH_2_	1.52, 1.70,* m*	26.1, CH_2_	1.52, 1.70,* m*
3	87.4, CH	3.00, *m*	87.5, CH	3.00, *m*	87.5, CH	3.00, *m*
4	37.2, qC		37.0, qC		37.2, qC	
5	55.7, CH	0.69, *m*	55.7, CH	0.69, *m*	55.5, CH	0.69, *m*
6	17.8, CH_2_	1.09, 1.45, *m*	17.8, CH_2_	1.09, 1.45, *m*	17.8, CH_2_	1.09, 1.45, *m*
7	35.5, CH_2_	1.36, 1.44, *m*	35.5, CH_2_	1.36, 1.44, *m*	35.5, CH_2_	1.36, 1.44, *m*
8	36.8, qC		36.9, qC		36.8, qC	
9	52.3, CH	0.80, *s*	52.3, CH	0.80, *s*	52.3, CH	0.80, *s*
10	36.8, qC		36.9, qC		36.8, qC	
11	22.6, CH_2_	1.33, 1.49, *m*	22.6, CH_2_	1.33, 1.49, *m*	22.6, CH_2_	1.33, 1.49, *m*
12	27.8, CH_2_	1.56, 1.72, *m*	27.9, CH_2_	1.56, 1.72, *m*	27.9, CH_2_	1.56, 1.72, *m*
13	36.1, CH	1.01, *m*	36.0, CH	1.01, *m*	36.1, CH	1.01, *m*
14	52.7, qC		52.9, qC		52.7, qC	
15	36.2, CH_2_	1.89, 2.38, *s*	36.0, CH_2_	1.89, 2.38, *s*	36.0, CH_2_	1.89, 2.38, *s*
16	109.5, qC		109.7, qC		109.3, qC	
17	53.1, CH	0.84, *s*	53.0, CH	0.84, *s*	53.0, CH	0.84, *s*
18	18.9, CH_3_	1.03, *s*	18.9, CH_3_	1.03, *s*	18.9, CH_3_	1.03, *s*
19	16.5, CH_3_	0.77, *s*	16.6, CH_3_	0.77, *s*	16.5, CH_3_	0.77, *s*
20	67.5, qC		67.7, qC		67.5, qC	
21	29.7, CH_3_	1.03, *s*	29.7, CH_3_	1.03, *s*	29.7, CH_3_	1.03, *s*
22	44.4, CH_2_	1.24, 1.36, *m*	44.4, CH_2_	1.24, 1.36, *m*	44.5, CH_2_	1.24, 1.36, *m*
23	67.4, CH	4.48, *m*	67.4, CH	4.48, *m*	67.4, CH	4.48, *m*
24	126.1, CH	5.08, *d,* (8.0)	126.1, CH	5.08, *d,* (8.0)	126.3, CH	5.08, *d,* (8,0)
25	133.6, qC		133.8, qC		133.6, qC	
26	18.6, CH_3_	1.60,* s*	18.6, CH_3_	1.60,* s*	18.6, CH_3_	1.60,* s*
27	25.7, CH_3_	1.65, *s*	25.7, CH_3_	1.65, *s*	25.8, CH_3_	1.65, *s*
28	27.6, CH_3_	0.89, *s*	27.6, CH_3_	0.89, *s*	27.6, CH_3_	0.89, *s*
29	16.4, CH_3_	0.68, *s*	16.4, CH_3_	0.68, *s*	16.4, CH_3_	0.68, *s*
30	65.4, CH_2_	3.41, 3.67, *s*	65.4, CH_2_	3.41, 3.67, *s*	65.3, CH_2_	3.41, 3.67, *s*

*qC* quaternary, *CH* methine, *CH*_*2*_ methylene, *CH*_*3*_ methyl carbons.

**Table 2:** DEPT-Q (400 MHz) and ^1^H (100 MHz) NMR data for the sugar moieties and attached acids of compounds **1, 2, 3** in DMSO-*d*_*6*_; Carbon multiplicities were determined by the DEPT-Q experiments.

Moiety	Position	1		2		3	
		^*δ*^_C_	^*δ*^_H_ (*J* in Hz)	^*δ*^_C_	^*δ*^_H_ (*J* in Hz)	^*δ*^_C_	^*δ*^_H_ (*J* in Hz)
Arab	1ˋ	104.2, CH	4.26, *d* (6.2)	104.6, CH	4.26, *d* (6.2)	104.3, CH	4.26, *d* (6.2)
	2ˋ	71.5, CH	3.73,* m*	71.5, CH	3.73,* m*	71.5, CH	3.73,* m*
	3ˋ	82.7, CH	3.70, *m*	82.7, CH	3.70, *m*	82.3, CH	3.70, *m*
	4ˋ	67.9, CH	3.87, *m*	67.9, CH	3.87, *m*	67.6, CH	3.87, *m*
	5ˋ	65.1, CH_2_	3.68, 3.39	65.1, CH_2_	3.68, 3.39	64.7, CH_2_	3.68, 3.39
Fuc	1ˋˋ	100.3, CH	5.32, *s*	100.7, CH	5.32, *s*	100.5, CH	5.32, *s*
	2ˋˋ	70.6, CH	3.61, *m*	70.6, CH	3.61, *m*	70.6, CH	3.61, *m*
	3ˋˋ	65.7, CH	3.60, *m*	65.7, CH	3.60, *m*	65.3, CH	3.60, *m*
	4ˋˋ	72.4, CH	3.60, *m*	72.4, CH	3.60, *m*	72.4, CH	3.60, *m*
	5ˋˋ	66.8, CH	4.11, *m*	66.8, CH	4.11, *m*	66.5, CH	4.11, *m*
	6ˋˋ	17.1, CH_3_	1.06,* d*, (6.0)	17.1, CH_3_	1.06,* d*, (6.0)	17.1, CH_3_	1.06,* d*, (6.0)
Glu	1ˋˋˋ	103.4, CH	4.32, *d*, (8.0)	103.8, CH	4.32, *d*, (8.0)	103.5, CH	4.32, *d*, (8.0)
	2ˋˋˋ	73.7, CH	3.51, *m*	73.2, CH	3.51, *m*	73.2, CH	3.51, *m*
	3ˋˋˋ	74.7, CH	3.86, *m*	75.0, CH	3.86, *m*	77.1, CH	3.86, *m*
	4ˋˋˋ	74.4, CH	4.62, *m*	74.0, CH	4.65, *m*	70.0, CH	3.50, *m*
	5ˋˋˋ	74.2, CH	3.50, *m*	74.6, CH	3.50, *m*	77.0, CH	3.50, *m*
	6ˋˋˋ	61.2, CH_2_	3.58, 3.70, *m*	61.2, CH_2_	3.58, 3.70, *m*	60.8, CH_2_	3.58, 3.70, *m*
Coumaroyl	1ˋˋˋˋ	125.5, qC		125.9, qC			
	2ˋˋˋˋ, 6ˋˋˋˋ	133.6, CH	7.59, *d,* (8.0)	130.4, CH	7.56, *d,* (8.0)		
	3ˋˋˋˋ, 5ˋˋˋˋ	115.9, CH	6.78, *d,* (8.0)	115.0, CH	6.81, *d,* (8.0)		
	4ˋˋˋˋ	159.0, qC		165.1, qC			
	7ˋˋˋˋ	145.2, CH	7.70, *d,* (16.0)	143.8, CH	7.70, *d,* (16.0)		
	8ˋˋˋˋ	114.2, CH	6.35, *d,* (16.0)	115.3, CH	6.35, *d,* (16.0)		
	9ˋˋˋˋ	164.6, qC		166.0, qC			
Oxaloyl	1ˋˋˋˋˋ			159.0, qC			
	2ˋˋˋˋˋ			174.7, qC			

*qC* quaternary, *CH* methine, *CH*_*2*_ methylene, *CH*_*3*_ methyl carbons, *Glc β*-D-glucopyranosyl, *Ara α*-L-arabinopyranosyl, *Fuc β*-D-fucopyranosyl.

Christinin F (2): white powder; [UV (MeOH) *λ*_max_ (log_ε_) 282 (5.5), 273 (6.0), 306 (4.5) nm; IR υ_max_ (KBr) 3570, 3540, 3400, 3100, 1730, 1350, 1300, 1000 cm^−1^; NMR data; see Tables 1 and 2; HRESIMS m/z 1131.5398 [M + H]^+^ (calc. for C_58_H_83_O_22_, 1131.5378).

### Acid_-_hydrolysis and sugar^-^analysis

Sugar hydrolysis and GC–MS^-^analysis of derivatives were carried out in accordance with Abbet et al. 2011^53^. Hydrolysis was performed individually on compounds 1 (1.0 mg), 2 (1.5 mg), and 3 (1.7 mg). The mixture was extracted with CH_2_Cl_2_ (3 × 1.0 mL) after^-^heating_-_at 100 °C for 1 h., in 2 M^-^TFA (1 mL). The aquoes phase was^-^freeze-dried^-^before being_-_redissolved in 200 mL of dry_-_pyridine with 5 mg/mL^-^L-cysteine methyl^-^ester hydrochloride. The reaction^-^mixture was heated^-^at 60 °C for 1 h., before^-^silylation with hexamethyldisilazane^-^and^-^chlorotrimethylsilane (Fluka) in^-^pyridine (3:1:10,^-^300 mL) for 30 min., at^-^60 °C^54^. Pyridine^-^was evaporated^-^after^-^silylation, and the^-^solid residue was^-^extracted with *n*-Hex. A 5890^-^Series II gas^-^chromatograph linked to an_-_HP 5971A^-^mass detector (Hewlett^-^Packard,^-^USA) was used for the^-^GC–MS analysis. The separation was performed on a^-^DB-225 MS^-^column (30 m, 0.25 mm,-I.D., Waters,^-^USA); column_-_temperature was 150 °C for 2 min., followed by a gradient of 58^−^C/min. to 210 °C, followed by a gradient of 10 °C/min. to 240 °C. When the retention-durations of derivatized_-_reference sugars were compared to those obtained from samples, L-arabinose (Rt 24.48 min.), D-glucose (Rt 28.64 min.), and D-fucose (Rt 25.96 min.) were found in all examined substances.

### Cell cultures

A549, U87, MDA-MB-231, and CT-26 cells were purchased from American Type Culture Collection (ATCC) (Manassas, VA, USA). The cells, A549 and CT-26 were cultured in RPMI-1640 supplemented with 1% Pen/Strep (100 U mL^–1^, Gibco, Carlsbad, CA) and 10% FBS (Atlanta Biologicals, Flowery Branch, GA). While MDA-MB-231 and U87 cells were cultured in DMEM medium supplemented with 1% Pen/Strip and 10% FBS. Cells were incubated at 37 °C and 5% CO_2_ in humidified incubator (Sanyo Scientific Autoflow, Hudson, MA).

### Cell viability testing using MTS

A549, U87, MDA-MB-231, and CT-26 cancer cells were plated in 96-well plates at a density of 2X10^4^ cells/well in 100 μL of medium before the addition of different concentrations of the plant extracts. Untreated control group was incubated with^-^100 µL/well of fresh media and^-^methanol. After 24 h., the medium was aspirated and replaced by_-_100 µL of fresh media, and 20 µL of MTS^-^reagent in each well (CellTiter 96^-^Aqueous One^-^Solution cell^-^proliferation assay, Promega^-^Corporation, Madison,^-^WI,^-^USA). The plates were then incubated at 37 °C with 5% CO_2_ for 2 h. The absorbance was measured at 490 nm using a Spectramax plus 384 Microplate reader (Molecular Devices, Sunnyvale, CA, USA). Relative cell viability values were expressed as the percentage of the absorbance from the treated wells compared to the control wells (untreated), given that the control wells viability was set to 100%. The cells were examined under a cell imaging system (EVOS FL Digital Microscope) using a 20X objective as well before getting rid of the plates. The half-maximal inhibitory concentrations (IC_50_) of pure compounds for four human cancer cell lines were calculated.

### In silico-investigation

#### Prediction-of-the-potential-targets

The exact isomeric^-^structure of saponins aglycone was generated by ChemDraw^55^, and then it was uploaded to Pharm Mapper platform (http://www.lilab-ecust.cn/pharmmapper/)^56^ in order to be inversely docked against the proteins hosted in the platform.

#### Targets of human cancer

Human cancer-relevant target proteins (Table S1) were collected from the following three databases: Gene Cards (https://www.genecards.org/)^57^; Therapeutic Target Database (TTD, http://db.idrblab.net/ttd/)^58^, Comparative Toxicogenomics Database (CTD, http://ctdbase.org/) and Drug Bank database (https://www.drugbank.ca/)^59^. The word “cancer” was selected as the keyword, and the species were limited as “*Homo sapiens*”. The targets that repeated at least two times were selected.

### Molecular docking MD simulation and network construction

Docking was performed using Aut^-^Dock Vina_-_software, MD^-^simulations were performed^-^using NAMD 3.0.0 software, and PPI network building was performed using cytoscape. The companion file contains detailed descriptions of these processes.

### Statistical analysis

The data were tabulated using the statistical programme-GraphPad-Prism-version-9 (GraphPad,-La-Jolla,-CA,-USA).

## Results and discussion

### Phytochemical investigation of *Ziziphus spina-christi*

Sugar moieties in saponins **1, 2,** and **3**, were identified by GC–MS after acid hydrolysis followed by derivatization with L-cystein methyl ester, subsequent silylation, and comparison with derivatized reference sugars.

**Figure 1:**
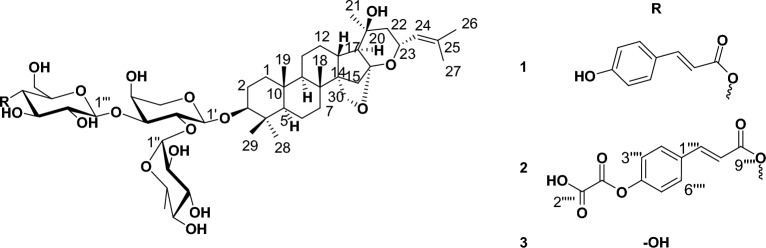
Structures of compounds isolated from *Ziziphus spina-christi*.

Based on_-_the physicochemical, chromatographic-properties, the-spectral-analyses-from UV, ^1^H, and-DEPT-Q-NMR, as well as-comparisons-with the-literature, the crude extract of *Z. spina-christi* leaves afforded the new saponins **1–2**, along with the known saponins christinin A **3** (C_47_H_77_O_17_, 913.5161, 10 degrees of unsaturation), which had been previously isolated from *Z. spinachristi* leaves^35^ (Tables 1, 2, Fig. 1).

**Figure 2:**
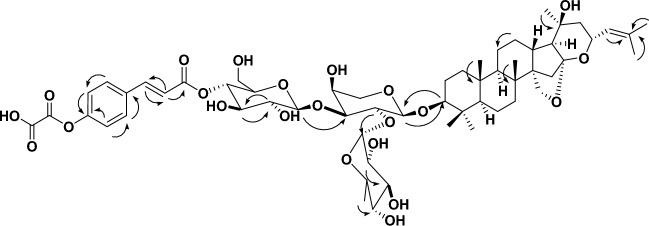
Selected **HMBC** () correlations of compound **2**.

The positive ion HRESIMS spectrum of **1** showed a signal at *m/z* 1059.5559 [M + H]^+^ corresponding to a molecular formula of C_56_H_83_O_19_ (calcd for C_56_H_83_O_19_, 1059.5529), suggesting 16 degrees of unsaturation. ^1^H and ^13^C NMR spectra of **1** were virtually superimposable to those of **3**, with the addition of four aromatic methine groups at *δ*_H_ 7.59 (2H, d, *J* = 8) *δ*_C_ 133.6, *δ*_H_ 6.78 (2H, d, *J* = 8) *δ*_C_ 115.9, two *trans*-olefinic methine groups at *δ*_H_ 7.70 (1H, d, *J* = 16) *δ*_C_ 145.2, *δ*_H_ 6.35 (1H, d, *J* = 16) *δ*_C_ 114.2, and three quaternary carbons at *δ*_C_ 125.5, 159.0, 164.6. These signals are suggestive characteristics for *trans*-*p*-coumaroyl moiety, reflecting the additional 146 mass units, and 6 degrees of unsaturation increase observed for **1** versus **3**. The signals corresponding to the CH-4ˋˋˋof the *β-*D*-*glucopyranosyl moiety (*δ*_H_ 4.62, *δ*_C_ 74.4) appeared downfield compared to those in **3** (*δ*_H_ 3.50, *δ*_C_ 70.0), while the signals corresponding to the CH-3ˋˋˋ, 5ˋˋˋ of the *β-*D*-*glucopyranosyl moiety (*δ*_H_ 3.86, 3.50 *δ*_C_ 74.7, 74.2) appeared upfield compared to those in **3** (*δ*_H_ 3.86, 3.50, *δ*_C_ 77.1, 77.0), and thereby indicated attachment of the *trans*-*p*-coumaroyl moiety at CH-4ˋˋˋof the *β-*D*-*glucopyranosyl moiety. The ^3^* J*-HMBC correlations of the proton H-4ˋˋˋ (*δ*_H_ 4.62, *δ*_C_ 74.0) with the quaternary carbonyl carbon C-9ˋˋˋˋ (*δ*_C_ 165.5), confirming the connections of the *trans*-*p*-coumaroyl moiety at this position (Table 2, Figs. 1, 2). Consequently, saponin **1** was identified as christinin E (Fig. 1).

The positive ion HRESIMS spectrum of **2** showed a signal at *m/z* 1131.5398 [M + H]^+^ corresponding to a molecular formula of C_58_H_83_O_22_ (calcd for C_58_H_83_O_22_, 1131.5378), suggesting 18 degrees of unsaturation. ^1^H and ^13^C NMR spectra of **2** were virtually superimposable to those of **1**, with the addition of two signals attributable to carboxylic groups in the DEPT-Q spectrum (*δ*_C_ 159.0, and 174.7), hence, the saponin was esterified with an oxaloyl moiety, reflecting the additional 71.98 mass units, and 2 degrees of unsaturation increase observed for **2** versus **1**. The signals corresponding to the C-4ˋˋˋˋ of the *trans*-*p*-coumaroyl moiety (*δ*_C_ 165.1) appeared downfield compared to those in **1** (*δ*_C_ 159.0), while the signals corresponding to the CH-2ˋˋˋˋ, 6ˋˋˋˋ & CH-3ˋˋˋˋ, 5ˋˋˋˋ of the *trans*-*p*-coumaroyl moiety (*δ*_H_ 7.56, 6.81*δ*_C_ 130.4, 115.0) appeared upfield compared to those in **1** (*δ*_H_ 7.59, 6.78, *δ*_C_ 133.6, 115.9), and thereby indicated attachment of the oxaloyl moiety at C-4ˋˋˋˋ in *trans*-*p*-coumaroyl moiety in **2**. Consequently, saponin **2** was identified as christinin F (Figs. 1, 2).

### Cytotoxic activity

The ability to make soap distinguishes steroidal or triterpenoid glycosides as saponins. Different saponins have been identified and purified, and their use in cancer chemotherapy is growing. High structural diversity in saponins is associated with anticancer effects such as reversing multidrug resistance (MDR), anti-metastasis, anti-proliferation, and anti-angiogenesis. These effects are brought about by the promotion of cell differentiation, bile acid–binding, induction of apoptosis, immune-modulatory effects, and amelioration of carcinogen-induced cell proliferation^60^. Consequently, different molecular mechanisms are contribute to saponins' anticancer action. It should be noted that the aglycone moiety, the length and linkage of the glycosidic chain, the presence of a functional carboxylic group on the aglycone chain, the number of sugar molecules and hydroxyl group, the position of the hydroxyl group, stereo-selectivity, and the type of sugar molecule on the glycine chain are all structural components that have a significant impact on the mechanism of saponins' anticancer action^61–63^.

**Figure 3:**
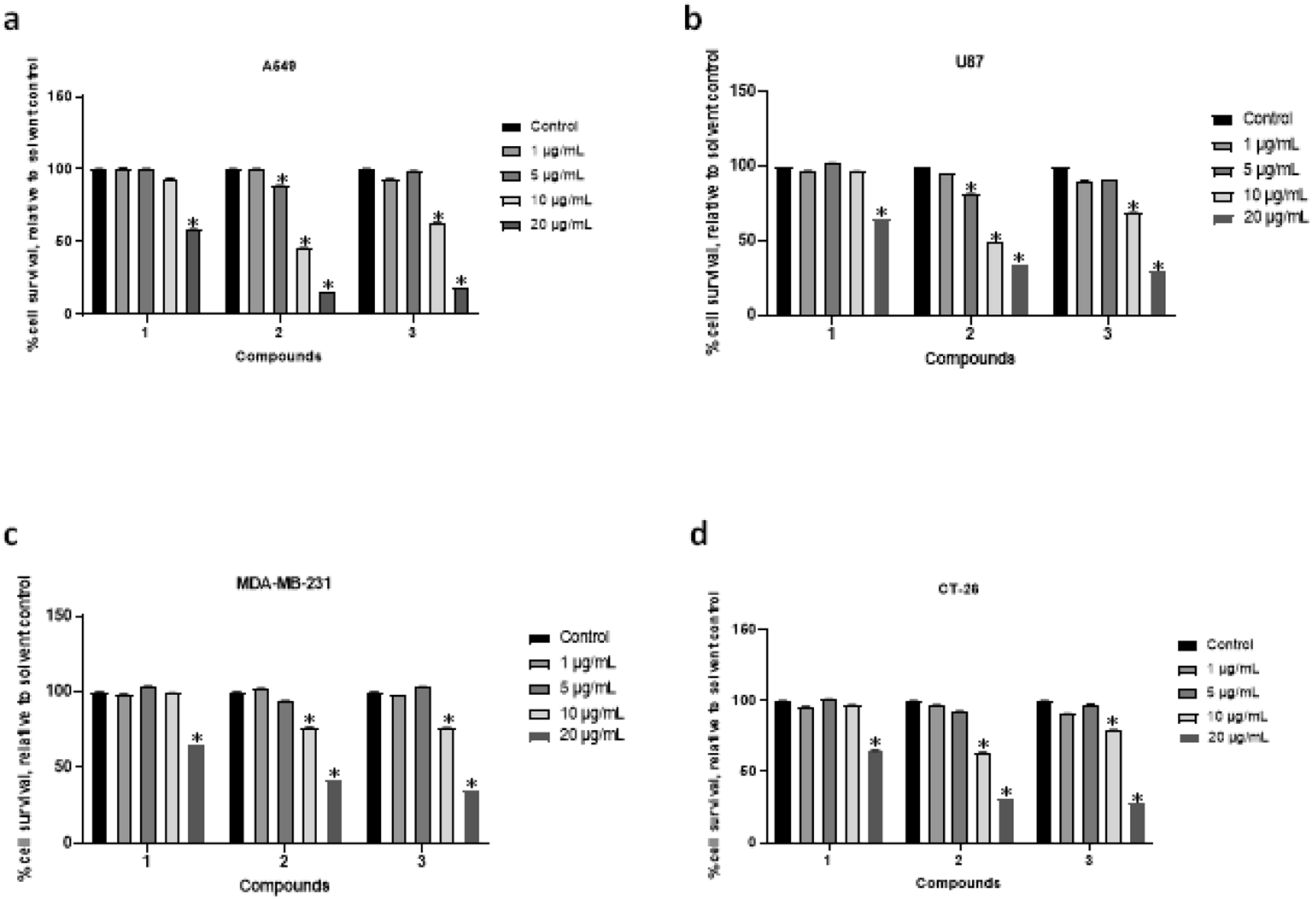
The anticancer activities of the three pure compounds (**1**–**3**) isolated from *Ziziphus spina-christi* using the MTS assay on glioblastoma, breast cancer, lung, and colorectal carcinoma cells. Cancer cells lines A549 (**a**) U87 (**b**), MDA-MB-231 (**c**) and CT-26 (**b**) were tested. Cells (2 × 10^4^ cells per well in 96 well plates) were treated with pure compounds as using the following concentrations (1, 5, 10 or 20 μg/mL) or a matching solvent control for 24 h at 37 °C with 5% CO_2_. Cells were stained with MTS reagents and cell survival was quantitated using the spectramax plate reader. The graphs represent the analysis of six replicates and show the percentage of survival of A549, U87, MDA-MB-231 and CT-26–- treated cells relative to matching solvent-treated cells. The solvent used is methanol. All experiments were performed at least two times. *p < 0.05, Student’s *t*-test.

In this investigation, the isolated dammarane type saponins compounds **1**–**3** from the methanolic extract of *Ziziphus spina-christi* leaves were evaluated for their cytotoxic activities against A549, U87, MDA-MB-231, and CT-26 cancer cell lines, using MTS. The results showed that three pure compounds exhibit a high anticancer efficacy, as evidenced by their ability to significantly reduce cancer cell viability. Notably, these compounds were strongly active at concentrations below 10 μg/mL, resulting in a 50% cytotoxic effect (Table 3, Fig. 3).

**Table 3:** The half-maximal inhibitory concentrations (IC_50_) of the three pure compounds (**1–3**) isolated from *Ziziphus spina-christi* on four human cancer cell lines.

Cell lines	IC_50_ (µg/mL) values		
	1	2	3
A549	9.53 ± 0.01	4.89 ± 0.93	5.67 ± 1.05
CT-26	0.70 ± 0.049	7.21 ± 0.35	5.47 ± 1.3
MDA-MB-231	6.02 ± 1.5	5.81 ± 0.087	9.3 ± 0.19
U87	5.70 ± 1.63	4.16 ± 0.87	7.28 ± 0.56

GraphPad Prism 9 software was used to calculate the half maximal inhibitory concentration IC_**50**_ (µg/mL) values. The concentration of each pure compound was transformed to log10. All experiments were performed at least two times. *p < 0.05, Student’s *t*-test.

Dammarane-type saponins gained attention after multiple articles demonstrated their anticancer activity against a range of malignancies. Gypenosides CP2, 5, 6, 7, 11, 12, 13, and 15 were identified and characterised from *Gynostemma pentaphyllum* aerial parts and demonstrated antiproliferative activities against two human tumour cell lines (A549 and HepG2)^64^. The EC_50_ values of these eight compounds against HepG2 cells ranged from 29.3 to 100.6 μM, while only four compounds (2, 11, 13, and 15) had EC_50_ values less than 100 μM (EC_50_ 59.487.3 μM) against A549 cells. Compound **2** was the most effective against A549 cells, whereas Compound **5** was among the most effective against HepG2 cells^64^. Another dammarane-type saponin, gypenoside Jh1, isolated from heat-processed *G. pentaphyllum* ethanol extract, exhibited strong cytotoxicity against A549 cells in a concentration-dependent manner, which was associated with apoptotic cell death as evidenced by morphological changes, Hoechst 33,258 nuclear staining, Annexin V and propidium iodide binding, and mitochondrial potentials assay^65^. New dammarane triterpene saponins called bacopaside É and bacopaside VII that were extracted from the n-BuOH fraction of *Bacopa monniera* demonstrated cytotoxicity against human tumour cell lines MDA-MB-231, SHG-44, HCT-8, A-549, and PC-3 M. Furthermore, at a concentration of 50 μmol/l, both compounds markedly reduced the adhesion, migration, and matrigel invasion of the breast cancer cell line MDA-MB-231 in vitro. These compounds at 50 μmol/kg demonstrated 90.52% and 84.13% inhibition in mice implanted with sarcoma S180 in in vivo studies^66^.

In a recent study, 20(R)-20-methoxyl-dammarane-3β,12β,25-triol, one of the novel dammarane-type sapogenins derived from *Panax ginseng* berries, demonstrated antiproliferative action against HepG2, Colon205, and HL-60 tumour cell lines, with IC_50_ values of 8.78, 8.64, and 3.98 mM, respectively^67^. From the heat-processed leaves of *P. ginseng*, other intriguing novel dammarane-type glycosides, specifically ginsenosides SL1–SL3, and 11 recognised chemicals were identified^68^. Ginsenosides Rh3 and Rk2, with IC_50_ values of 0.8 and 0.9 mM, respectively, had strong actions against human leukaemia HL-60 cells. Furthermore, ginsenosides SL3, 20S-Rg2, F4, and 20S-Rh2 showed promising action, with corresponding IC_50_ values of 9.0, 9.0, 7.5, and 8.2 mM. Notably, there have been reports of anticancer efficacy when ginsenosides such Rh2, Rg1, and Rg3 are administered orally^69–71^. Two novel dammarane monodesmosides, centellosides A and B, have been found in whole *Centella asiatica* plants. These plants exhibited modest in vitro cytotoxicity against HepG2 and K562 cells, according to a recent phytochemical investigation^72^.

In human cancer cell lines, panaxadiol, a protopanaxadiol-type ginsenoside with a dammarane structure, also showed anticancer effects when combined with cyclophosphamide, 5-fluorouracil, and epicatechinin^73,74^. Strong anticancer activity has been observed for three derivatives of panaxadiol: 3β-acetoxy-panaxadiol, 3β-palmitic acid aceloxy-panaxadiol, and 3β-octadecanoic-panaxadiol^75^. High inhibitory effects against EBV-EA were demonstrated by dammarenolic acid, with IC_50_ values of 226 mol ratio/32 pmol TPA. Ginsenosides are categorised as either dammarane-type or oleanane-type based on the differences in their aglycones. In the first group, it was demonstrated that 20(S)-ginsenoside Rg3 (Rg3) inhibited the proliferation of multiple tumour cells, including B16 melanoma cells, Lewis lung cancer cells, prostate cancer cells, and colon cancer cells^76^.

There are currently no known FDA-approved saponin-based anticancer medicines, despite the substantial research on saponins and their strong anticancer properties. This has a number of drawbacks, including toxicities and drug-like characteristics. In recent investigations, methods for ensuring enhanced efficacy and less toxicity in saponin were examined, including combination therapy and drug delivery systems^27^.

### Bioinformatics-based analysis

#### PPI network of the cancer related targets and KEGG-based enrichment analysis

The possible interactions between cancer-relevant proteins, first, all reported human-based proteins related to cancer disease, particularly for those tested in vitro in the present study (i.e., brain, breast, lung, colon cancers) were studied. Using each cancer type and “human”as keywords, we searched the Toxicogenomics (https://ctdbase.org/) and GeneCards databases, as well as the previously published literature. As a result, more than 1043 proteins relevant to these cancers were retrieved. Only the common proteins (551 proteins) for each cancer type were selected for the subsequent analysis (Table S1).

We utilized the Cytoscape^-^software to create a protein–protein^-^interaction (PPI) network from these 551 selected proteins.

**Figure 4:**
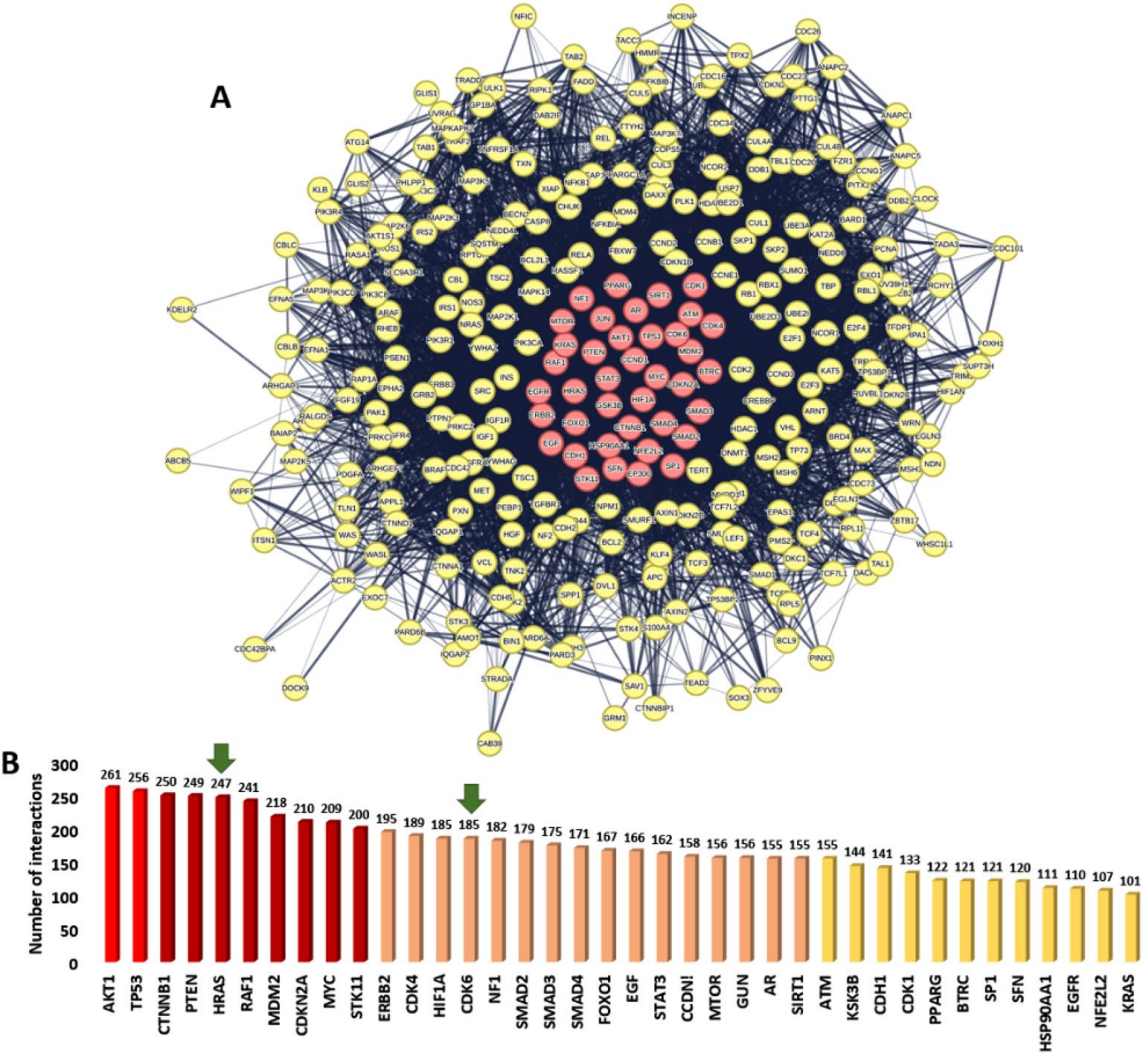
(**A**) Human cancer PPI network. This network comprises 306 nodes and 7602 edges with an average node degree of 49.7. The top-interacting nodes were colored by red (7.9%, 38 proteins of all interacting nodes, i.e., hub protein). (**B**) The top interacting-nodes (i.e., hub nodes arranged by their degree value). Green arrows represent the proteins predicted as probable targets for saponins aglycone investigated in the presented study. The thickness of the lines (i.e., edges) represents the degree of confidence (i.e., the strength of data support).

Figure 4A displays the network characteristics of the generated PPI, which included a high degree of connectivity (as seen by the 7602 edges between 306 nodes, a mean_-_node degree of 49.7, and a local clustering value of 0.557). The remaining selected proteins in 551 proteins list (Table S1) did not show any connections, so they were removed from the PPI network.

Therapeutic strategies for cancer may have a better chance of success if they focus on proteins having a high level of interaction, as these are typically the most significant and pertinent molecular targets (i.e., hub proteins or genes) in a given network^77^. Therefore, we focused on the top 8% (38 proteins) of the most intensively molecular targets that interact (i.e., hub proteins) sorted according to their degree value (Fig. 4B).

**Figure 5:**
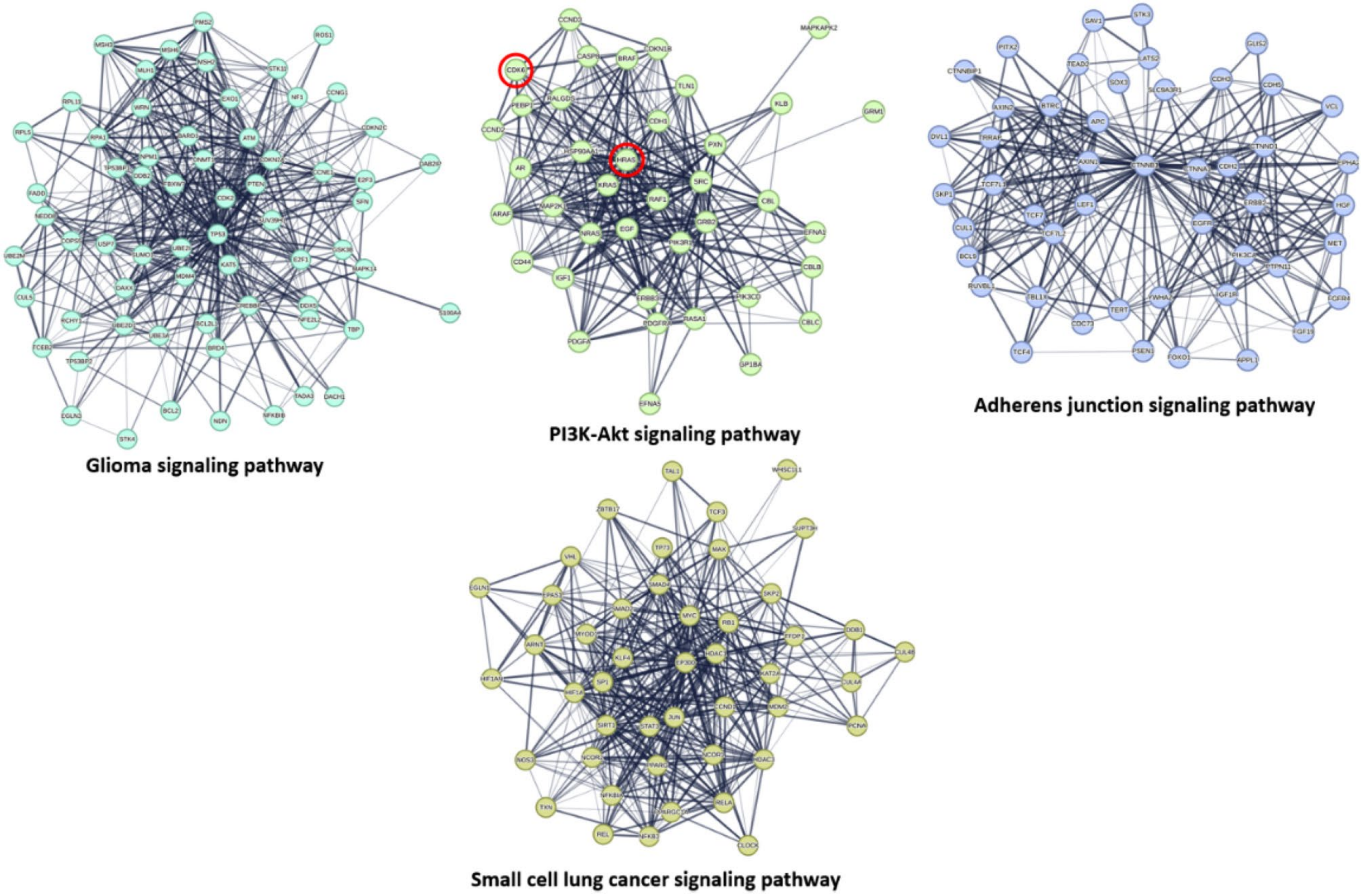
Subnetworks derived from the complete cancer-relevant network in Fig. 4. These networks represent the first 4 key pathways involved in cancer pathogenesis according to the presented PPI networking in Fig. 4. The thickness of the lines (i.e., edges) represents the degree of confidence (i.e., the strength of data support). Nodes encircled with red line in the PI3K-Akt signaling pathway cluster are CDK6 and HRAS that were predicted to be potential targets for compound **3**.

**Figure 6:**
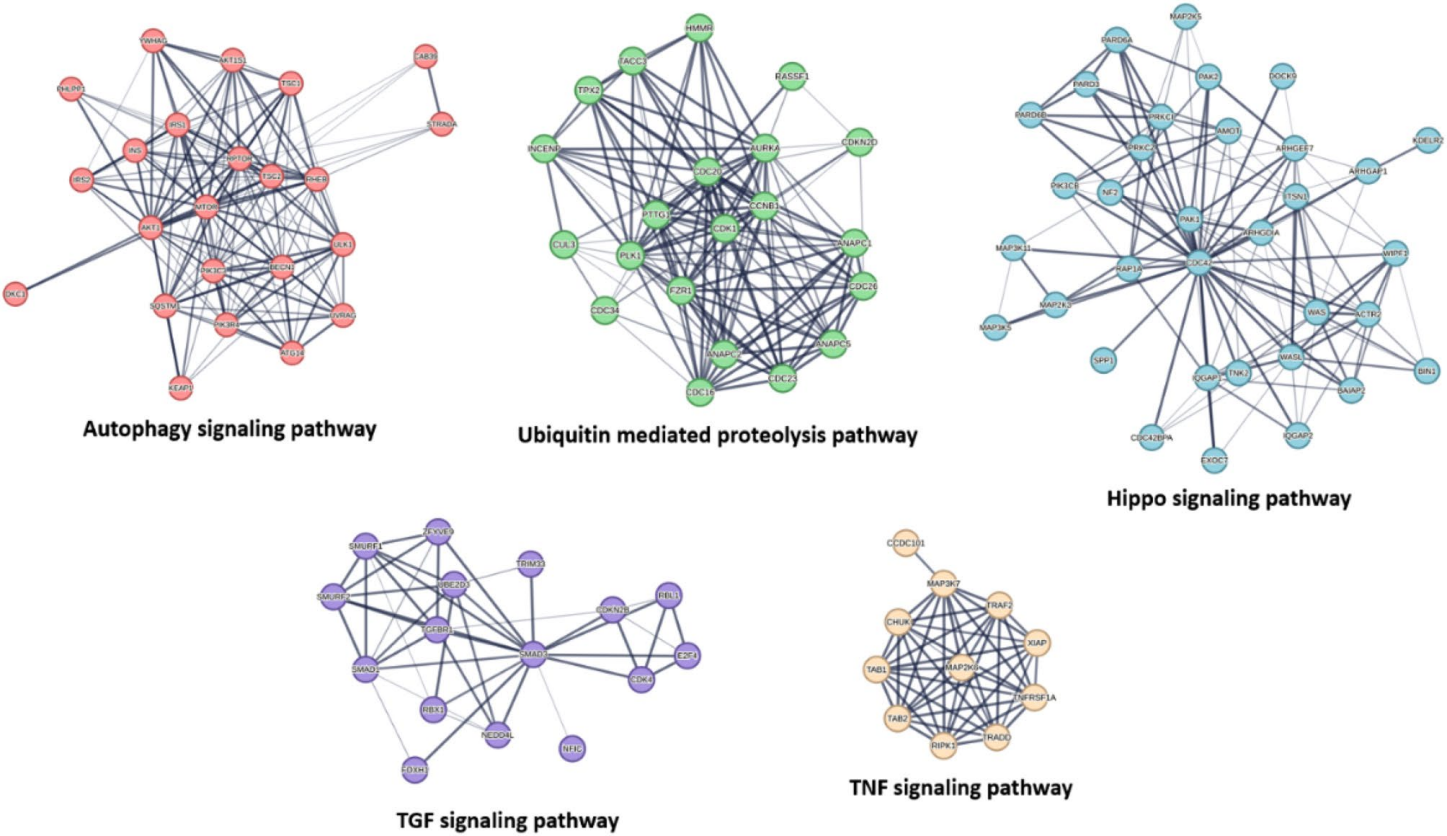
Subnetworks derived from the complete cancer-relevant network in Fig. 4. These networks represent the second 5 key pathways involved in the cancer pathogenesis according to the presented PPI networking in Fig. 4. The thickness of the lines (i.e., edges) represents the degree of confidence (i.e., the strength of data support).

In addition, We organised the proteins in the existing network based on their role in the various signaling pathways associated with cancer pathogenesis. The KEGG database (https://www.genome.jp/kegg/pathway.html) was used to guide this protein enrichment analysis. Proteins presented in the PPI network (Fig. 5) were categorized according to their involvement in the key cancer disease pathways into 9 groups: (i) Glioma signaling pathway; (ii) P3K-Akt signaling pathway; (iii) Adherens junction signaling pathway; (iv) Small cell lung cancer signaling pathway; (v) Autophagy signaling pathway; (vi) Ubiquitin mediated proteolysis pathway; (vii) Hippo signaling pathway; (viii) TGF signaling pathway; and (ix) TNF signaling pathway (Figs. 5, 6). Altogether, the current PPI network for human cancer offers a quick overview of the interacting proteins and associated signalling pathways, suggesting essential proteins that can be deemed critical to disease progression and thus suitable drug development targets.

#### Prediction of the target proteins for compounds 1–3

Multiple in silico-based experiments were subsequently performed on compounds **1–3** to putatively characterize its potential as an anticancer agent. The modeled structures of compounds **1–3** were run through the PharmMapper (http://www.lilab-ecust.cn/pharmmapper/) prediction platform to see if they might bind to any cancer-relevant protein target. PharmMapper is a unique pharmacophore-based virtual screening platform that can match the query structure into the 3D active sites-derived pharmacophore maps of most proteins hosted in the protein data bank (PDB; https://www.rcsb.org/)^56^.

Unfortunately, none of the three compounds showed interactions with any cancer-related protein with an acceptable score (Fit score > 4). Even direct docking of the structures of these compounds to the cancer-relevant hub-proteins (Fig. 4) did not retrieve significant interaction with acceptable scores (< − 5 kcal/mol). These negative findings could be attributed to the large structure and the hydrophilic nature of these compounds that made them undruggable structures.

The bioactivity of triterpenoidal and steroidal glycosides is usually linked to their aglycone part^78–81^ because these glycosides can be easily hydrolyzed either spontaneously or in acidic conditions like this found in the stomach^82,83^. Hence, the aglycon parts of these glycosides are the key players in the bioactivity of such molecules e.g., glycyrrhetinic acid and diosgenin are the aglycones of both glycyrrhizin and dioscin, respectively. Both aglycones have been found to be the active anticancer molecules^79,84^.

Accordingly, we used the aglycone of the three compounds (**1**–**3**) for our *in-silico* analysis to link these major metabolites and their cancer-relevant target(s). So, the modeled structure of saponins aglycone was subjected to the PharmMapper-based pharmacophore screening to find out the potential cancer-relevant protein targets for this structure. Four proteins (i.e., BDR3, GLO1, CDK6, and HRAS) were predicted as potential targets for saponins aglycone only of which two proteins (i.e., CDK6, and HRAS) have been identified as hub proteins in the cancer PPI network described above (Fig. 4). Additionally, both proteins were involved in the PI3K-Akt signaling pathway (Fig. 5), indicating that saponins aglycone probably can kill cancer cells via this signaling pathway.

**Figure 7:**
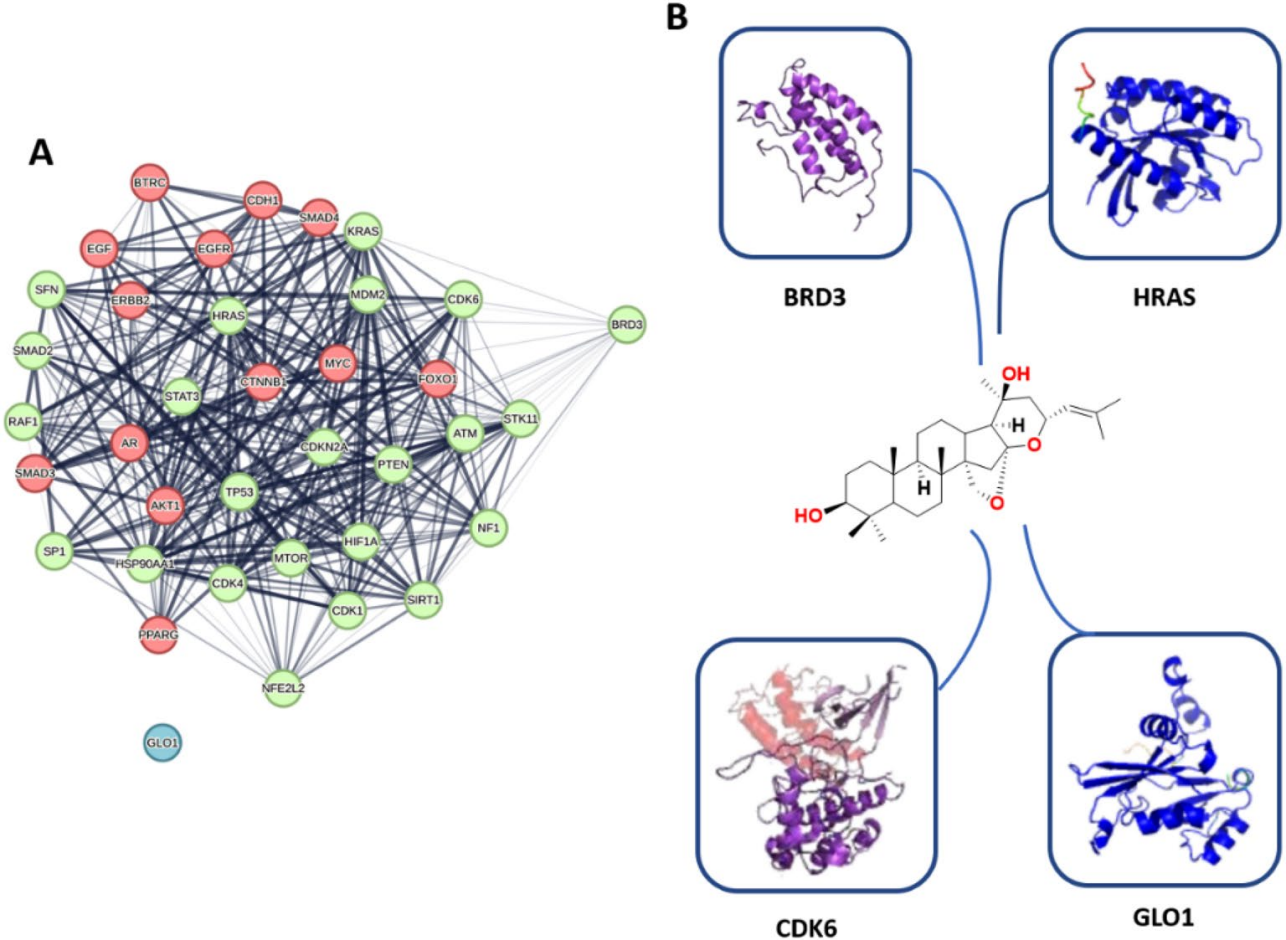
(**A**) PPI network showing the interactions among the 38 hub proteins characterized in the complete cancer PPI network in Fig. 4 including both GLO1 and BDR3 that were identified as a probable target for saponins aglycone. Lemon-green-colored nodes represent proteins that show interactions with BDR3, while the red-colored ones are not. GLO1 (the blue-colored node) do not show any interactions. (**B**) The structures of saponins aglycone along with the structures of the four proteins predicted to be potential target for it.

By generating another PPI network for the previously identified hub proteins (i.e., 38 highly interacting proteins in the network presented in Fig. 4) together with both GLO1 and BDR3, we were able to identify how the latter two proteins (i.e., BDR3 and GLO1) are essential in relation to these hub proteins. As shown in Fig. 7, BDR3 was found to interact with 25 hub proteins, while GLO1 did not show any interactions. This finding indicates that targeting BDR3 by saponins aglycone might also achieve a significant anticancer effect.

Saponin aglycones were then subjected to molecular docking and MD simulation experiments, which helped us to refine our preliminary pharmacophore-based virtual screening. Proteins were targets for saponins aglycone if they had docking scores with saponins aglycone below − 7 kcal/mol and absolute binding free energies (Δ*G*_binding_) below − 7 kcal/mol. Accordingly, all the predicted proteins followed these conditions (Table S2), and hence, subjected to further refinement in 100 ns-long MD simulations.

#### Analysis saponins aglycone’s modes of interaction

To investigate the binding modes of saponins aglycone inside the active site of each predicted protein, its modeled structure was re-docked into these active sites using Autodock Vina, and then the top-scoring binding pose for each protein was inspected and subjected to 100 ns-long MD simulation to check for the binding stability od saponins aglycone inside the active site of each protein.

**Figure 8:**
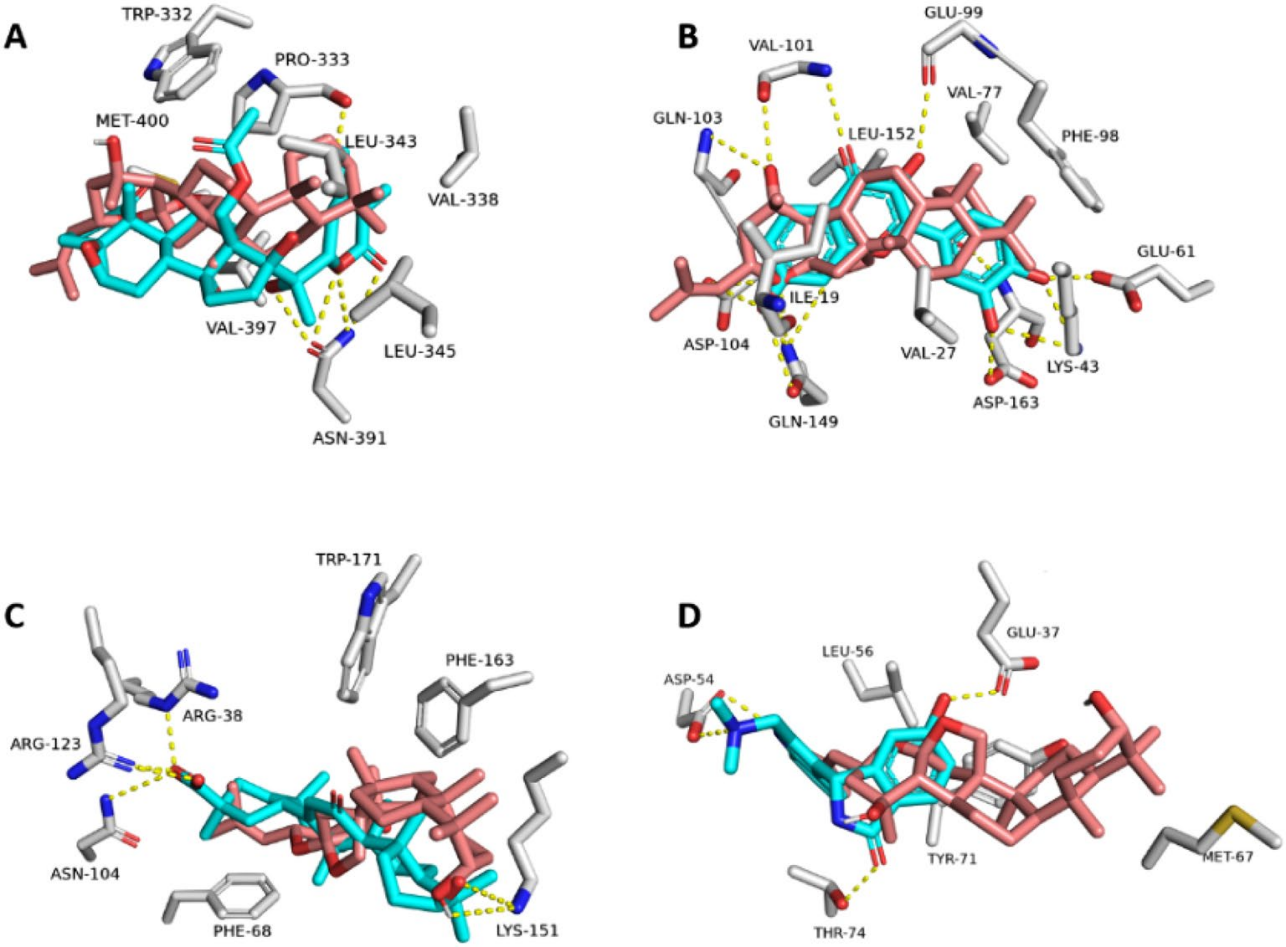
Binding modes of saponins aglycone (brick red-colored structure) in alignment with the co-crystalized inhibitors (cyan-colored structures) inside BDR3, CDK6, GLO1, and HRAS ((**A**–**D**), respectively).

Saponin aglycones were found to bind inside the active sites of BDR3, GLO1, CDK6, and HRAS (PDB IDs: 7S3P, 4PV5, 1XO2, and 6ZL3, respectively) with molecular interactions comparable to the co-crystallized ligands (Fig. 8).

**Figure 9:**
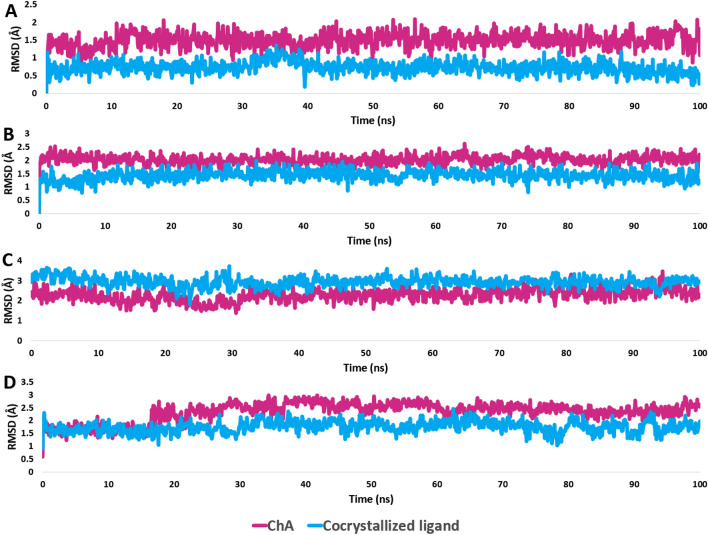
RMSDs of saponins aglycone and the co-crystalized inhibitors inside BDR3, CDK6, GLO1, and HRAS ((**A**–**D**), respectively) over the course of 100 ns-long MD simulations.

First, BRD3: saponins aglycone and the co-crystallized ligand were found to be in a good alignment, and hence, were able to form stable binds within the enzyme's active site, with modest fluctuations and average RMSDs of 1.22 Å and 1.78 Å, as shown by 100 ns of MD simulations (Fig. 9A and B). Both saponins aglycone and the co-crystallized ligand shared the same hydrophobic interactions with TRP-332, PRO-333, VAL-338, LEU-343, LEU-345, and VAL-397, while differed in the H-bond formation, where saponins aglycone established single H-bond with the main chain of PRO-333, and the co-crystallized ligand formed 4 H-bonds with ASN-391. BDR3 (Bromodomain-containing protein 3) is a small globular protein that has been found to play a key role in chromatin remodeling and act as an epigenetic modifier^85,86^. Depletion or inhibition of BRD3 slows the growth of a number of cancer cell lines, including prostate and glioblastoma cell lines^87,88^. Recently, the withanolide physachenolide C (BDR3’s co-crystalized ligand) has been reported to potently inhibit BDR3^89^. physachenolide C is a steroidal derivative, and hence, similar derivatives like saponins aglycone could be a good alternative inhibitor for this important cancer-relevant protein.

Second, GLO1: saponins aglycone and the co-crystallized ligand (i.e., 18-β-glycyrrhetinic acid; a triterpene derivative like saponins aglycone) were found to be in a good alignment. Accordingly, they were able to form similar interactions within the enzyme's active site (Fig. 9C). Both saponins aglycone and the co-crystallized ligand shared the same hydrophobic interactions with PHE-68, LYS-151, PHE-163, and TRP-171, while differed in the H-bond formation, where saponins aglycone established two H-bond with the LYS-151, and the co-crystallized ligand formed 3 H-bonds with ARG-38, ASN-104, and ARG-123 (Fig. 8C). Regarding their binding stability, both saponins aglycone and the co-crystallized inhibitor exhibited almost the same fluctuations and RMSD profiles (average RMSD = 2.34 Å and 2.84 Å, respectively) over the course of 100 ns of MD simulations (Fig. 9C).

GLO1 (glyoxalase I) catalyzes the conversion of hemimercaptal, formed from methylglyoxal and glutathione, to S-lactoylglutathione, and is involved in the regulation of TNF-induced transcriptional activity of NF-kappa-B^90^. GLO1 is overexpressed in cancer cells in response to the redox state, and hence, it is related to multi-drug resistance in chemotherapy, making GLOI modulators and/or inhibitors as potential anticancer therapeutics^91^.

Third, CDK6: saponins aglycone and the co-crystallized ligand (i.e., fisetin) were in a comparable alignment. Accordingly, they were able to form similar interactions within the enzyme's active site (Fig. 8C). However, the flavanol derivative fisetin (i.e., the co-crystalized inhibitor) established more H-bonds due to its multiple hydroxyl groups. Both saponins aglycone and the co-crystallized ligand shared the same hydrophobic interactions with ILE-19, VAL-27, VAL-77, PHE-98, and LEU-152, while differed in the H-bond formation, where saponins aglycone established two H-bond with the main chains of VAL-101 and GLN-103, and the polyhydroxylated co-crystallized inhibitor formed 8 H-bonds with LYS-43, GLU-61, GLU-99, ASP-163, and ASP-104 (Fig. 8C). About their binding stability upon simulation, both saponins aglycone and the co-crystallized inhibitor exhibited comparable fluctuations and RMSD profiles (average RMSD = 1.87 Å and 2.49 Å, respectively) over the course of 100 ns of MD simulations (Fig. 9C).

CDK6 (cyclin-dependent kinase 6) CDK6 is involved in a positive feedback loop that activates transcription factors through a reaction cascade. Importantly, these C-CDK complexes act as a kinase, phosphorylating and inactivating the protein of Rb and p-Rb related “pocket proteins” p107 and p130. While doing this, the CDK6 in conjunction with CDK4, act as a switch signal that first appears in G1, directing the cell towards S phase of the cell cycle^92,93^. CDK6 is overexpressed in cancer cells, and hence, it has been considered a key target for the development of efficient anticancer agents^94^.

Finally, HRAS: saponins aglycone and the co-crystallized ligand were not fully aligned with each other. (Fig. 8C). Hence, they shared only the same hydrophobic interactions with LEU-56 and TYR-71, while saponins aglycone did not form any H-bonds inside the active site of the enzyme (Fig. 8C). However, it was able to establish stable binding comparable to the co-crystallized inhibitor over the course of the MD simulation with an acceptable deviation (average RMSD = 2.66 Å and 1.99 Å, for saponins aglycone and the co-crystallized inhibitor, respectively) (Fig. 9D).

HRAS (Harvey rat sarcoma viral oncogene homolog) binds GDP/GTP and possesses intrinsic GTPase activity that is involved in the activation of Ras protein signal transduction^95^. This oncoprotein has been proven to be associated with a wide variety of tumors, and hence, targeting and inhibiting this protein is an excellent strategy to develop efficient anticancer medications. Until recently, there were not previously described HRAS inhibitors (similar to the remaining RAS oncoproteins). In 2020, several new potent HRAS inhibitors were reported targeting an allosteric binding site^96^, which in the present study, was also predicted to be a probable binding site for saponins aglycone.

From the previous network pharmacology-based and in silico-based findings, it can be concluded that saponins aglycone is a promising scaffold for the development of novel and efficient anticancer agents that can act via multiple targets and signaling pathways. Accordingly, such a comprehensive bioinformatics-based investigation should be the primary step in the development of new therapeutics, particularly from natural products.

On the other hand, it should be emphasized that such in-silico analysis presented herein has some limitations and assumptions that need to be considered when interpreting the results. First, the docking methods used to predict the binding poses and affinities of the compounds are based on empirical scoring functions that may not accurately capture the complex interactions and dynamics of the protein–ligand system. Second, the docking methods do not account for the effects of solvation, entropy, and conformational changes on the binding free energy. Third, the docking methods rely on the availability and quality of the protein structures, which may not reflect the native conformation or flexibility of the target. Therefore, the obtained in silico based-results should be regarded as tentative hypotheses that need to be validated by experimental methods.

## Conclusion

The findings of this study demonstrated that three pure dammarane type saponins compounds which identified as christinin A, E, and F, and, derived from *Z. spina-christi* leaf extract, exhibited anti-cancer efficacy againt A549, U87, MDA-MB-231, and CT-26 cell lines. Notably, these compounds were strongly active at concentrations below 10 μg/mL, resulting in a 50% cytotoxic effect. Our results showed that all compounds exhibited low IC_50_ values, indicating their potency at low concentrations across all four cancer cell lines. A PPI network analysis was used to identify 17 potential targets of saponins aglycone in cancer cells, and the binding modes of saponins aglycone to four of these targets (BRD3, GLO1, CDK6, and HRAS) were confirmed using docking and MD simulations. It was found that saponins aglycone could bind stably and similarly to the co-crystallized ligands of these proteins, and modulate their functions and affect their cancer-related pathways. As a result, anticancer effects such as cell cycle arrest, apoptosis, DNA damage, and inhibition of cell migration and invasion were induced by saponins aglycone. These observations alongside those obtained from the comprehensive network pharmacology-based and i*n silico-*based analyses suggest that these compounds, particularly their aglycone, hold promise as potential candidates for cancer treatment. However, further investigations are necessary to elucidate the cellular mechanism of action for these compounds. Beside, in vivo studies to validate the cytotoxic effects of the isolated saponins, as well as an evaluation of their toxicity and potential side effects.

### Supplementary Information

Supplementary Information.

## Data Availability

Partial data generated or analysed during this study are included in this published article, and its supplementary information files.
