# Supplementary material for: New cytotoxic dammarane type saponins from Ziziphus spina-christi
Source: Sci Rep. 2023 Nov 23;13:20612. doi: 10.1038/s41598-023-46841-2 (PMC10667233; doi:10.1038/s41598-023-46841-2)
Supplement: Supplementary file 1 — Supplementary Information. [file 41598_2023_46841_MOESM1_ESM.docx]

**SUPPLEMENTARY DATA**

**New Cytotoxic Dammarane Type Saponins from *Ziziphus spina-christi***

Abeer H. Elmaidomy^1^, Amr EL Zawily^2,3*^, Aliasger K Salem^3^, Faisal H. Altemani^4^, Naseh A. Algehainy^4^, Abdullah H. Altemani^5^, Mostafa E. Rateb^6^, Usama Ramadan Abdelmohsen^7,8*,^ Nourhan Hisham Shady^8^

^1^Department of Pharmacognosy, Faculty of Pharmacy, Beni-Suef University, Beni-Suef 62511, Egypt

^2^ Department of Plant and Microbiology, Faculty of Science, Damanhour University, Damanhour, 22511, Egypt

^3^ Division of Pharmaceutics and Translational Therapeutics, College of Pharmacy, University of Iowa, Iowa City 52242, IA, USA; Salem, aliasger-salem@uiowa.edu

^4^ Department of Medical Laboratory Technology, Faculty of Applied Medical Sciences, University of Tabuk, Tabuk 71491, Saudi Arabia. faltemani@ut.edu.sa; nalgehainy@ut.edu.sa

^5^Department of Family and Community Medicine, Faculty of Medicine, University of Tabuk, Tabuk 71491, Saudi Arabia, atemani@ut.edu.sa

^6^ School of Computing, Engineering & Physical Sciences, University of the West of Scotland, Paisley PA1 2BE, UK; mostafa.rateb@uws.ac.uk

^7^ Department of Pharmacognosy, Faculty of Pharmacy, Minia University, Minia 61519, Egypt

^8^ Department of Pharmacognosy, Faculty of Pharmacy, Deraya University, Universities Zone, New Minia 61111, Egypt; norhan.shady@deraya.edu.eg

Corresponding: [usama.ramadan@mu.edu.eg](mailto:usama.ramadan@mu.edu.eg), [amr-elzawily@uiowa.edu](mailto:amr-elzawily@uiowa.edu)


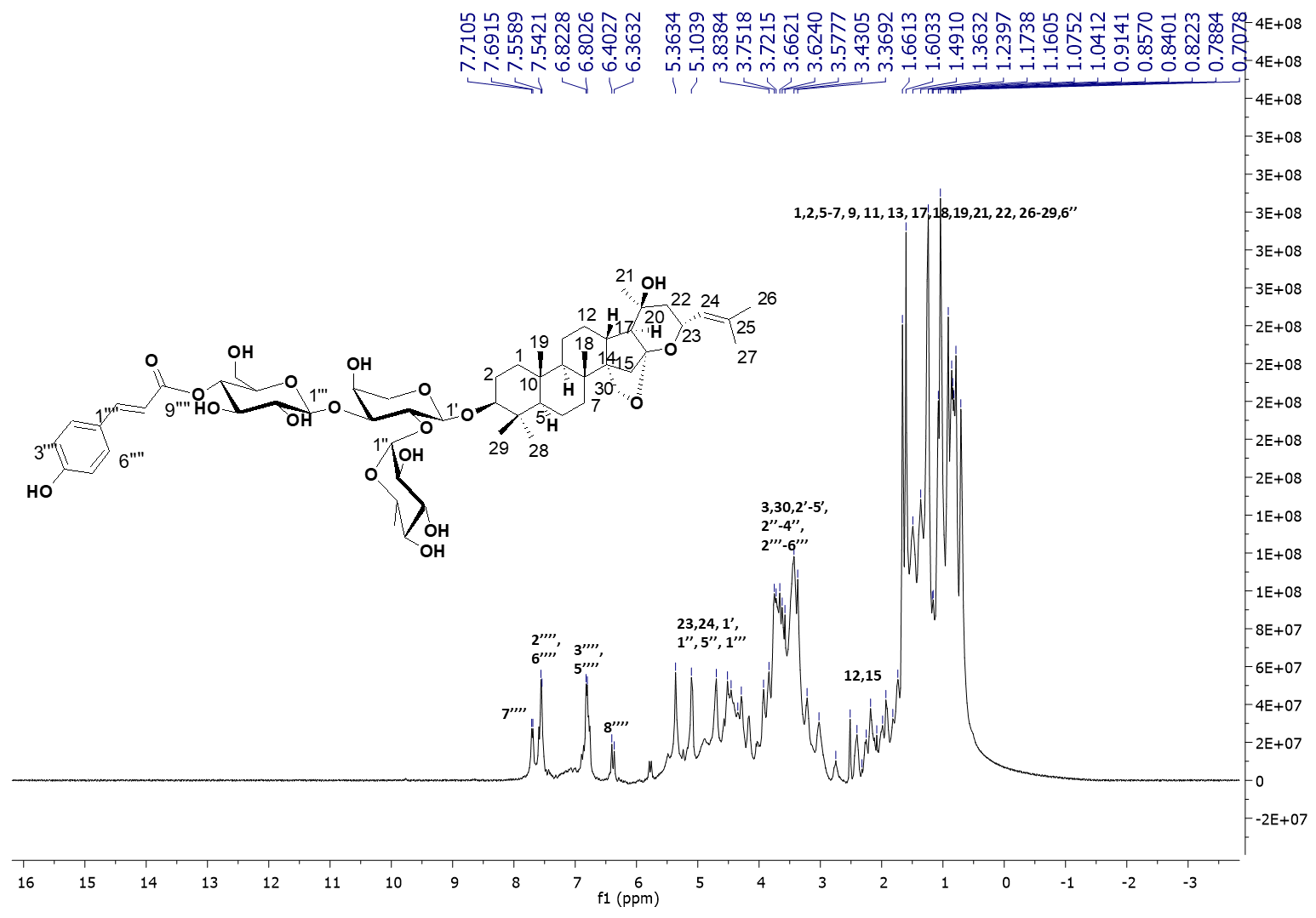


**Figure S1.** ^1^H NMR spectrum of compound **1** measured in DMSO-*d_6_* at 400 MHz


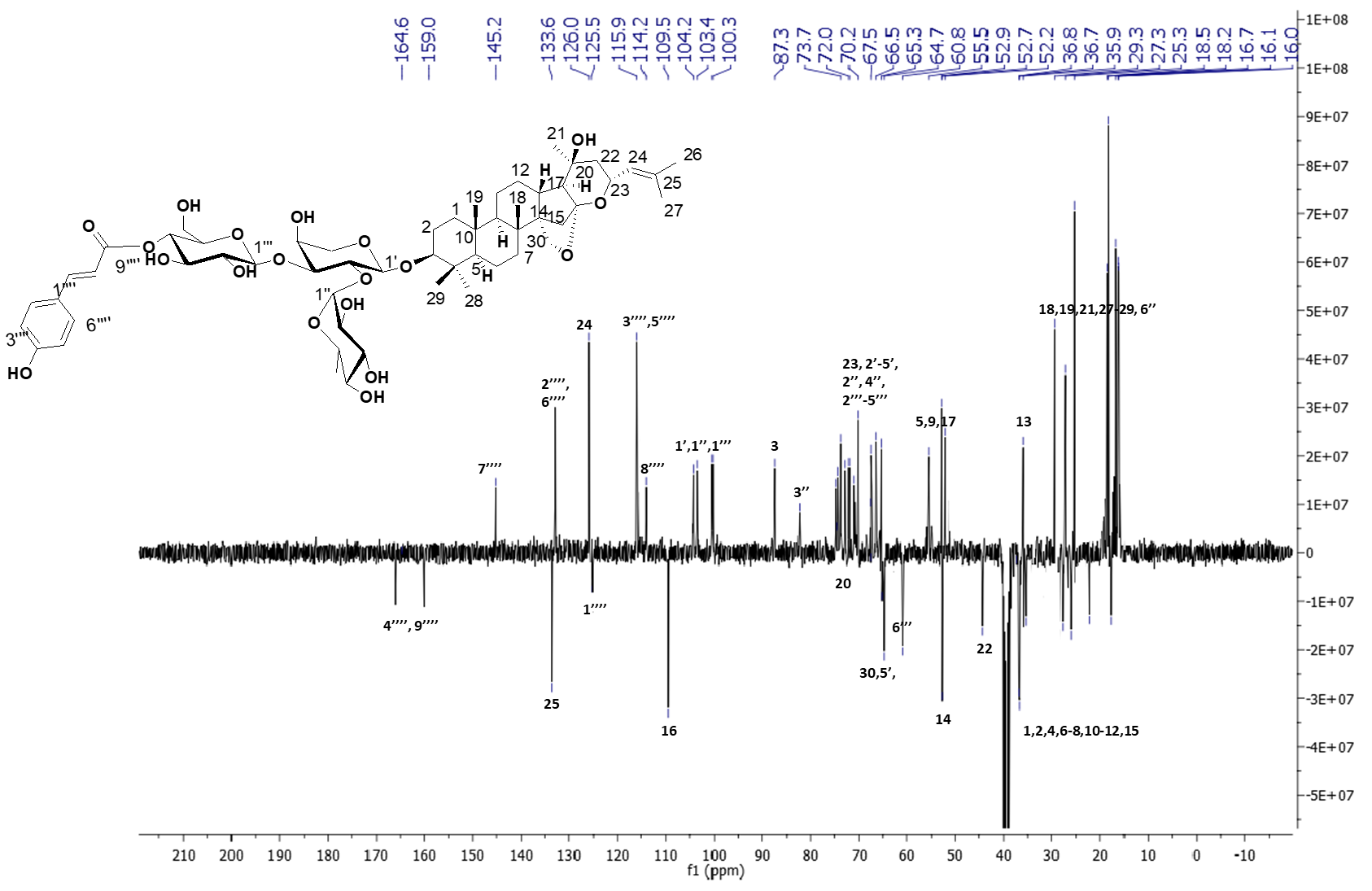


**Figure S2.** DEPT-Q NMR spectrum of compound **1** measured in DMSO-*d_6_* at 100 MHz


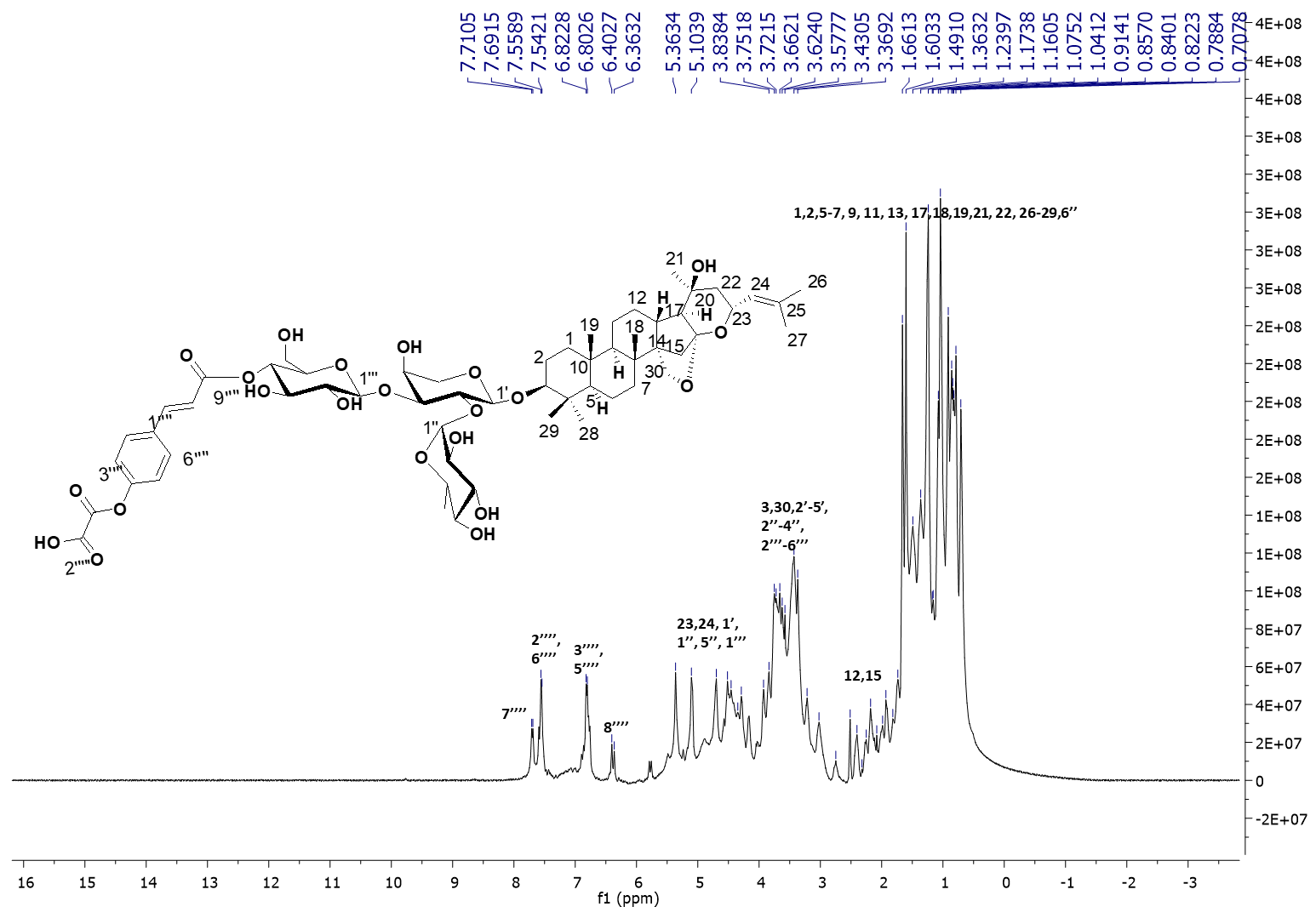


**Figure S3.** ^1^H NMR spectrum of compound **2** measured in DMSO-*d_6_* at 400 MHz


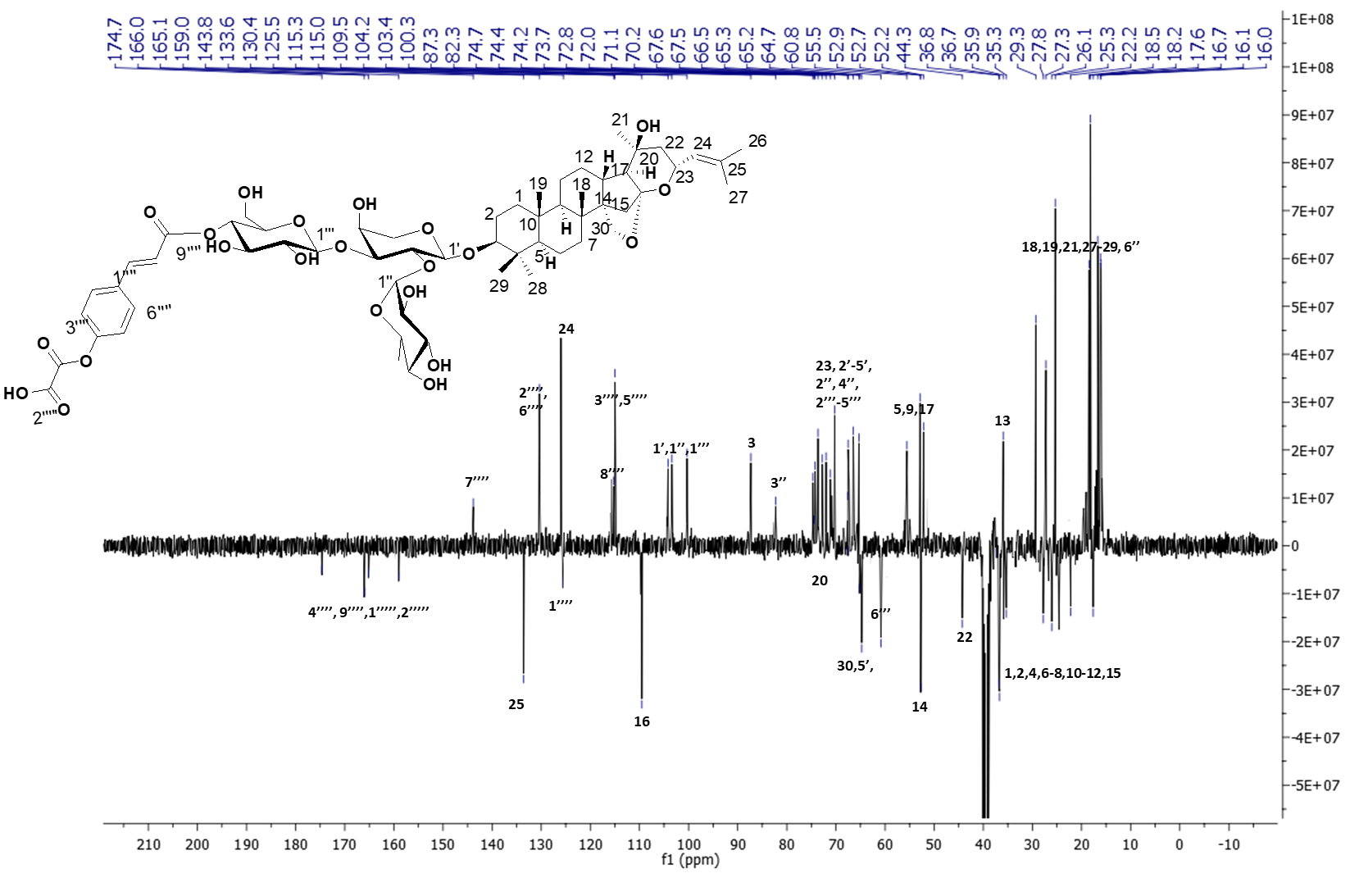


**Figure S4.** DEPT-Q NMR spectrum of compound **2** measured in DMSO-*d_6_* at 100 MHz

**
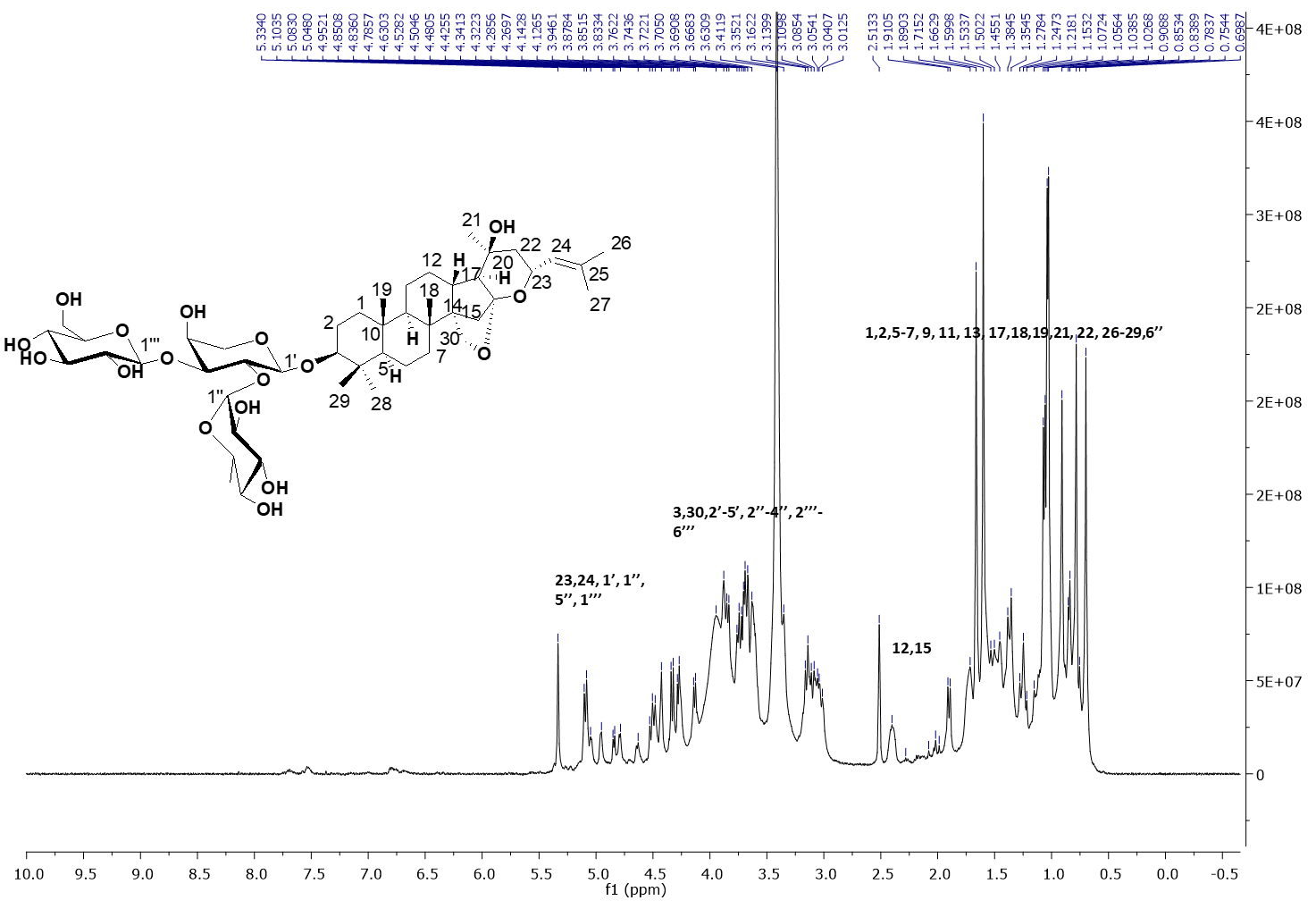
**

**Figure S5.** ^1^H NMR spectrum of compound **3** measured in DMSO-*d_6_* at 400 MHz


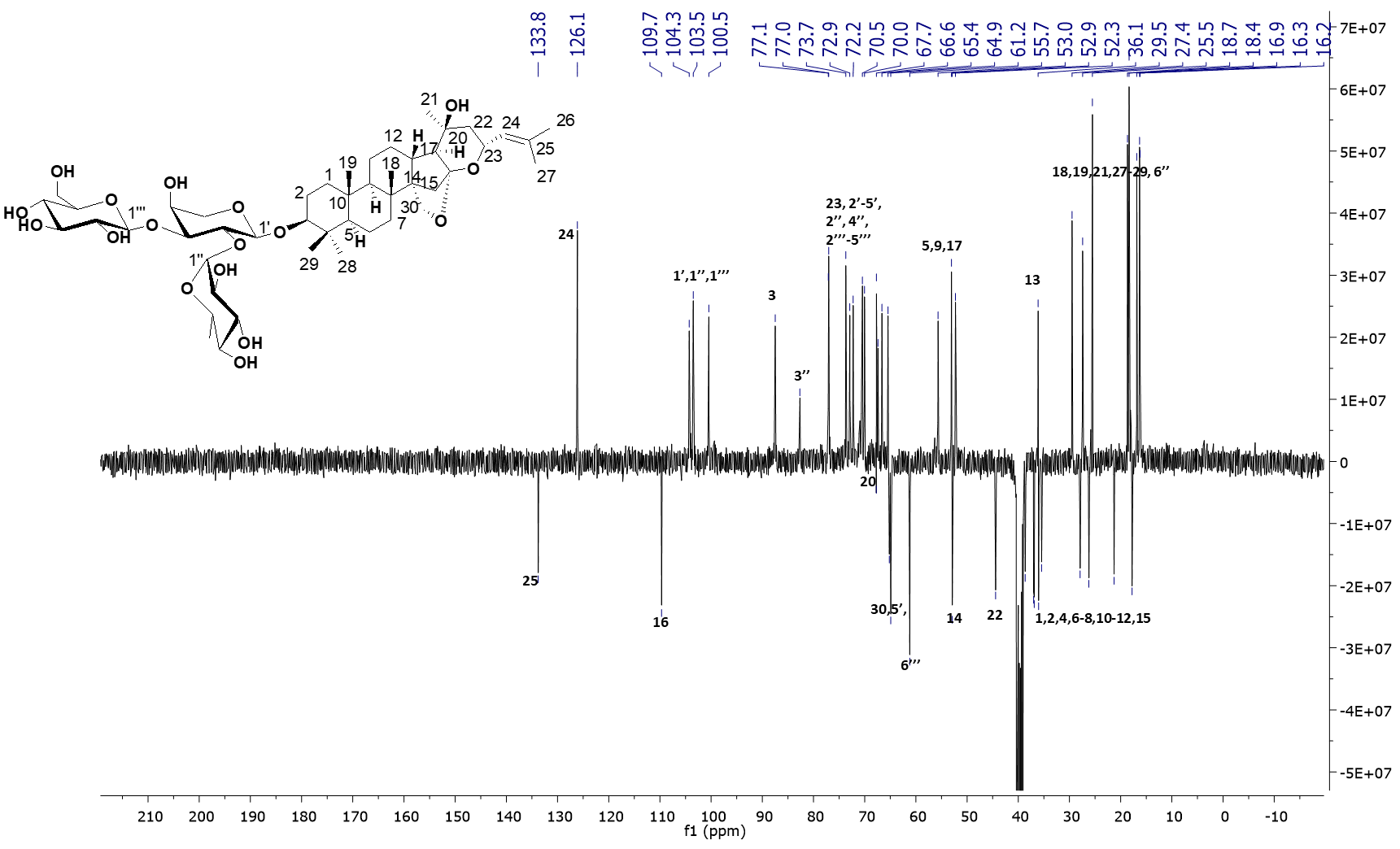


**Figure S6.** DEPT-Q NMR spectrum of compound **3** measured in DMSO-*d_6_* at 100 MHz

Table S1. Selected cancer-relevant targets that were used in the PPI network analysis.

| No. | Target |
| --- | --- |
| 1 | ABCB5 |
| 2 | AKT1 |
| 3 | AKT1S1 |
| 4 | ARNT |
| 5 | AURKA |
| 6 | CCND2 |
| 7 | CCNE1 |
| 8 | CD44 |
| 9 | CDC42 |
| 10 | CDK4 |
| 11 | CDK6 |
| 12 | CDKN2A |
| 13 | CDKN2B |
| 14 | CDKN2C |
| 15 | E2F1 |
| 16 | EPHA2 |
| 17 | ERBB2 |
| 18 | FGFR4 |
| 19 | FOXO1 |
| 20 | HIF1A |
| 21 | HRAS |
| 22 | IGF1R |
| 23 | KAT2A |
| 24 | KEAP1 |
| 25 | LATS2 |
| 26 | MAP2K3 |
| 27 | MAP3K5 |
| 28 | MAPK14 |
| 29 | MDM2 |
| 30 | MDM4 |
| 31 | MET |
| 32 | MYC |
| 33 | NF1 |
| 34 | NF2 |
| 35 | PDGFRA |
| 36 | PIK3R1 |
| 37 | PTEN |
| 38 | RAF1 |
| 39 | RB1 |
| 40 | RELA |
| 41 | SMAD2 |
| 42 | SOX3 |
| 43 | STK11 |
| 44 | STK3 |
| 45 | STK4 |
| 46 | TCF3 |
| 47 | TEAD2 |
| 48 | TERT |
| 49 | TNFRSF1A |
| 50 | TP53 |
| 51 | TSC1 |
| 52 | YWHAZ |
| 53 | AKT1 |
| 54 | YWHAZ |
| 55 | AKT1 |
| 56 | AKT1S1 |
| 57 | ARNT |
| 58 | AURKA |
| 59 | CCND2 |
| 60 | CCNE1 |
| 61 | CD44 |
| 62 | CDC42 |
| 63 | CDK4 |
| 64 | CDK6 |
| 65 | CDKN2A |
| 66 | CDKN2B |
| 67 | CDKN2C |
| 68 | E2F1 |
| 69 | EPHA2 |
| 70 | ERBB2 |
| 71 | FGFR4 |
| 72 | FOXO1 |
| 73 | HIF1A |
| 74 | HRAS |
| 75 | IGF1R |
| 76 | KAT2A |
| 77 | KEAP1 |
| 78 | LATS2 |
| 79 | MAP2K3 |
| 80 | MAP3K5 |
| 81 | MAPK14 |
| 82 | MDM2 |
| 83 | MDM4 |
| 84 | MET |
| 85 | MYC |
| 86 | NF1 |
| 87 | NF2 |
| 88 | PDGFRA |
| 89 | PIK3R1 |
| 90 | RELA |
| 91 | SMAD2 |
| 92 | STK11 |
| 93 | STK3 |
| 94 | STK4 |
| 95 | TEAD2 |
| 96 | TERT |
| 97 | TSC1 |
| 98 | YWHAZ |
| 99 | ABCB5 |
| 100 | AKT1 |
| 101 | AKT1S1 |
| 102 | ARAF |
| 103 | ARNT |
| 104 | AURKA |
| 105 | BECN1 |
| 106 | CBLB |
| 107 | CBLC |
| 108 | CCND2 |
| 109 | CCNE1 |
| 110 | CD44 |
| 111 | CDC42 |
| 112 | CDK4 |
| 113 | CDK6 |
| 114 | CDKN2A |
| 115 | CDKN2B |
| 116 | CDKN2C |
| 117 | CTNNB1 |
| 118 | DACH1 |
| 119 | E2F1 |
| 120 | EPHA2 |
| 121 | ERBB2 |
| 122 | FGFR4 |
| 123 | FOXO1 |
| 124 | FZR1 |
| 125 | GLIS1 |
| 126 | GLIS2 |
| 127 | GRM1 |
| 128 | HGF |
| 129 | HIF1A |
| 130 | HRAS |
| 131 | IGF1R |
| 132 | KAT2A |
| 133 | KDELR2 |
| 134 | KEAP1 |
| 135 | LATS2 |
| 136 | MAP2K3 |
| 137 | MAP2K5 |
| 138 | MAP2K6 |
| 139 | MAP3K5 |
| 140 | MAPK14 |
| 141 | MDM2 |
| 142 | MDM4 |
| 143 | MET |
| 144 | MSH2 |
| 145 | MYC |
| 146 | NF1 |
| 147 | NF2 |
| 148 | NFIC |
| 149 | PDGFRA |
| 150 | PIK3R1 |
| 151 | PTEN |
| 152 | RAF1 |
| 153 | RASSF1 |
| 154 | RB1 |
| 155 | RELA |
| 156 | ROS1 |
| 157 | SFN |
| 158 | SMAD1 |
| 159 | SMAD2 |
| 160 | SOX3 |
| 161 | STK11 |
| 162 | STK3 |
| 163 | STK4 |
| 164 | TCF3 |
| 165 | TEAD2 |
| 166 | TERT |
| 167 | TNFRSF1A |
| 168 | TP53 |
| 169 | TSC1 |
| 170 | TTYH2 |
| 171 | WHSC1L1 |
| 172 | YWHAG |
| 173 | YWHAZ |
| 174 | AKT1 |
| 175 | ARNT |
| 176 | AURKA |
| 177 | CCND2 |
| 178 | CCNE1 |
| 179 | CD44 |
| 180 | CDC42 |
| 181 | CDK4 |
| 182 | CDK6 |
| 183 | CDKN2A |
| 184 | CDKN2B |
| 185 | CDKN2C |
| 186 | E2F1 |
| 187 | EPHA2 |
| 188 | ERBB2 |
| 189 | FGFR4 |
| 190 | FOXO1 |
| 191 | HIF1A |
| 192 | KAT2A |
| 193 | KEAP1 |
| 194 | LATS2 |
| 195 | MAP3K5 |
| 196 | SMAD2 |
| 197 | STK11 |
| 198 | TEAD2 |
| 199 | TERT |
| 200 | TSC1 |
| 201 | YWHAZ |
| 202 | AKT1 |
| 203 | AURKA |
| 204 | CCNE1 |
| 205 | EPHA2 |
| 206 | FGFR4 |
| 207 | KEAP1 |
| 208 | LATS2 |
| 209 | MAP3K5 |
| 210 | SMAD2 |
| 211 | STK11 |
| 212 | TEAD2 |
| 213 | TERT |
| 214 | TSC1 |
| 215 | YWHAZ |
| 216 | ABCB5 |
| 217 | AKT1 |
| 218 | AKT1S1 |
| 219 | ARNT |
| 220 | AURKA |
| 221 | BECN1 |
| 222 | CCND2 |
| 223 | CCNE1 |
| 224 | CD44 |
| 225 | CDC42 |
| 226 | CDK4 |
| 227 | CDK6 |
| 228 | CDKN2A |
| 229 | CDKN2B |
| 230 | CDKN2C |
| 231 | E2F1 |
| 232 | EPHA2 |
| 233 | ERBB2 |
| 234 | FGFR4 |
| 235 | FOXO1 |
| 236 | HIF1A |
| 237 | HRAS |
| 238 | IGF1R |
| 239 | KAT2A |
| 240 | KEAP1 |
| 241 | LATS2 |
| 242 | MAP2K3 |
| 243 | MAP3K5 |
| 244 | MAPK14 |
| 245 | MDM2 |
| 246 | MDM4 |
| 247 | MET |
| 248 | MYC |
| 249 | NF1 |
| 250 | NF2 |
| 251 | PDGFRA |
| 252 | PIK3R1 |
| 253 | PTEN |
| 254 | RAF1 |
| 255 | RB1 |
| 256 | RELA |
| 257 | SMAD2 |
| 258 | SOX3 |
| 259 | STK11 |
| 260 | STK3 |
| 261 | STK4 |
| 262 | TCF3 |
| 263 | TEAD2 |
| 264 | TERT |
| 265 | TNFRSF1A |
| 266 | TP53 |
| 267 | TSC1 |
| 268 | YWHAZ |
| 269 | ABCB5 |
| 270 | AKT1 |
| 271 | AKT1S1 |
| 272 | ARAF |
| 273 | ARNT |
| 274 | AURKA |
| 275 | BECN1 |
| 276 | BRAF |
| 277 | CBLB |
| 278 | CBLC |
| 279 | CCND2 |
| 280 | CCNE1 |
| 281 | CD44 |
| 282 | CDC42 |
| 283 | CDK4 |
| 284 | CDK6 |
| 285 | CDKN2A |
| 286 | CDKN2B |
| 287 | CDKN2C |
| 288 | CTNNB1 |
| 289 | DACH1 |
| 290 | E2F1 |
| 291 | EPHA2 |
| 292 | ERBB2 |
| 293 | FGFR4 |
| 294 | FOXO1 |
| 295 | FZR1 |
| 296 | GLIS1 |
| 297 | GLIS2 |
| 298 | GRM1 |
| 299 | HGF |
| 300 | HIF1A |
| 301 | HRAS |
| 302 | IGF1R |
| 303 | KAT2A |
| 304 | KDELR2 |
| 305 | KEAP1 |
| 306 | LATS2 |
| 307 | MAP2K3 |
| 308 | MAP2K5 |
| 309 | MAP2K6 |
| 310 | MAP3K5 |
| 311 | MAPK14 |
| 312 | MDM2 |
| 313 | MDM4 |
| 314 | MET |
| 315 | MSH2 |
| 316 | MYC |
| 317 | NF1 |
| 318 | NF2 |
| 319 | NFIC |
| 320 | PDGFRA |
| 321 | PIK3R1 |
| 322 | PTEN |
| 323 | RAF1 |
| 324 | RASSF1 |
| 325 | RB1 |
| 326 | RELA |
| 327 | ROS1 |
| 328 | SFN |
| 329 | SMAD1 |
| 330 | SMAD2 |
| 331 | SOX3 |
| 332 | STK11 |
| 333 | STK3 |
| 334 | STK4 |
| 335 | TCF3 |
| 336 | TEAD2 |
| 337 | TERT |
| 338 | TNFRSF1A |
| 339 | TP53 |
| 340 | TSC1 |
| 341 | TTYH2 |
| 342 | WHSC1L1 |
| 343 | YWHAG |
| 344 | YWHAZ |
| 345 | ABCB5 |
| 346 | AKT1 |
| 347 | AKT1S1 |
| 348 | ARNT |
| 349 | AURKA |
| 350 | BECN1 |
| 351 | CBLB |
| 352 | CCND2 |
| 353 | CCNE1 |
| 354 | CD44 |
| 355 | CDC42 |
| 356 | CDK4 |
| 357 | CDK6 |
| 358 | CDKN2A |
| 359 | CDKN2B |
| 360 | CDKN2C |
| 361 | E2F1 |
| 362 | EPHA2 |
| 363 | ERBB2 |
| 364 | FGFR4 |
| 365 | FOXO1 |
| 366 | HIF1A |
| 367 | HRAS |
| 368 | IGF1R |
| 369 | KAT2A |
| 370 | KEAP1 |
| 371 | LATS2 |
| 372 | MAP2K3 |
| 373 | MAP3K5 |
| 374 | MAPK14 |
| 375 | MDM2 |
| 376 | MDM4 |
| 377 | MET |
| 378 | MYC |
| 379 | NF1 |
| 380 | NF2 |
| 381 | PDGFRA |
| 382 | PIK3R1 |
| 383 | PTEN |
| 384 | RAF1 |
| 385 | RB1 |
| 386 | RELA |
| 387 | SMAD2 |
| 388 | SOX3 |
| 389 | STK11 |
| 390 | STK3 |
| 391 | STK4 |
| 392 | TCF3 |
| 393 | TEAD2 |
| 394 | TERT |
| 395 | TNFRSF1A |
| 396 | TP53 |
| 397 | TSC1 |
| 398 | YWHAZ |
| 399 | ABCB5 |
| 400 | AKT1 |
| 401 | AKT1S1 |
| 402 | ARNT |
| 403 | AURKA |
| 404 | BECN1 |
| 405 | CBLB |
| 406 | CBLC |
| 407 | CCND2 |
| 408 | CCNE1 |
| 409 | CD44 |
| 410 | CDC42 |
| 411 | CDK4 |
| 412 | CDK6 |
| 413 | CDKN2A |
| 414 | CDKN2B |
| 415 | CDKN2C |
| 416 | E2F1 |
| 417 | EPHA2 |
| 418 | ERBB2 |
| 419 | FGFR4 |
| 420 | FOXO1 |
| 421 | HIF1A |
| 422 | HRAS |
| 423 | IGF1R |
| 424 | KAT2A |
| 425 | KEAP1 |
| 426 | LATS2 |
| 427 | MAP2K3 |
| 428 | MAP3K5 |
| 429 | MAPK14 |
| 430 | MDM2 |
| 431 | MDM4 |
| 432 | MET |
| 433 | MYC |
| 434 | NF1 |
| 435 | NF2 |
| 436 | PDGFRA |
| 437 | PIK3R1 |
| 438 | PTEN |
| 439 | RAF1 |
| 440 | RB1 |
| 441 | RELA |
| 442 | SMAD2 |
| 443 | SOX3 |
| 444 | STK11 |
| 445 | STK3 |
| 446 | STK4 |
| 447 | TCF3 |
| 448 | TEAD2 |
| 449 | TERT |
| 450 | TNFRSF1A |
| 451 | TP53 |
| 452 | TSC1 |
| 453 | YWHAZ |
| 454 | AKT1 |
| 455 | AURKA |
| 456 | CCND2 |
| 457 | CCNE1 |
| 458 | EPHA2 |
| 459 | FGFR4 |
| 460 | KEAP1 |
| 461 | LATS2 |
| 462 | MAP3K5 |
| 463 | SMAD2 |
| 464 | STK11 |
| 465 | TEAD2 |
| 466 | TERT |
| 467 | TSC1 |
| 468 | YWHAZ |
| 469 | AKT1 |
| 470 | CCNE1 |
| 471 | MAP2K3 |
| 472 | AKT1 |
| 473 | AURKA |
| 474 | CCND2 |
| 475 | CCNE1 |
| 476 | CD44 |
| 477 | EPHA2 |
| 478 | FGFR4 |
| 479 | KEAP1 |
| 480 | LATS2 |
| 481 | MAP3K5 |
| 482 | SMAD2 |
| 483 | STK11 |
| 484 | TEAD2 |
| 485 | TERT |
| 486 | TSC1 |
| 487 | YWHAZ |
| 488 | AKT1 |
| 489 | AURKA |
| 490 | CCND2 |
| 491 | CCNE1 |
| 492 | CD44 |
| 493 | CDC42 |
| 494 | EPHA2 |
| 495 | FGFR4 |
| 496 | KEAP1 |
| 497 | LATS2 |
| 498 | MAP3K5 |
| 499 | SMAD2 |
| 500 | STK11 |
| 501 | TEAD2 |
| 502 | TERT |
| 503 | TSC1 |
| 504 | YWHAZ |
| 505 | AKT1 |
| 506 | AURKA |
| 507 | CCND2 |
| 508 | CCNE1 |
| 509 | CD44 |
| 510 | CDC42 |
| 511 | CDK4 |
| 512 | EPHA2 |
| 513 | FGFR4 |
| 514 | KEAP1 |
| 515 | LATS2 |
| 516 | MAP3K5 |
| 517 | SMAD2 |
| 518 | STK11 |
| 519 | TEAD2 |
| 520 | TERT |
| 521 | TSC1 |
| 522 | YWHAZ |
| 523 | AKT1 |
| 524 | AURKA |
| 525 | CCND2 |
| 526 | CCNE1 |
| 527 | CD44 |
| 528 | CDC42 |
| 529 | CDK4 |
| 530 | CDK6 |
| 531 | EPHA2 |
| 532 | FGFR4 |
| 533 | KEAP1 |
| 534 | LATS2 |
| 535 | MAP3K5 |
| 536 | SMAD2 |
| 537 | STK11 |
| 538 | TEAD2 |
| 539 | TERT |
| 540 | TSC1 |
| 541 | YWHAZ |
| 542 | AKT1 |
| 543 | AURKA |
| 544 | CCND2 |
| 545 | CCNE1 |
| 546 | CD44 |
| 547 | CDC42 |
| 548 | CDK4 |
| 549 | CDK6 |

Table S2. The proteins that were predicted to be potential targets for ChA.

.

| **No.** | **Target** | **Full name** | **PDB ID** | **PharmMapper (Fit score score)** | **Vina score kcal/mol** | **ΔG_binding_**  **kcal/mol** |
| --- | --- | --- | --- | --- | --- | --- |
| 1 | BDR3 | Bromodomain-containing protein 3 | 7s3p | 8.67 | -9.32 | -8.11 |
| 2 | GLO1 | Glyoxalase I | 4pv5 | 8.38 | -8.43 | -7.91 |
| 3 | CDK6 | Cyclin-dependent kinase 6 | 1xo2 | 7.33 | -7.89 | -7.37 |
| 4 | HRAS | Harvey rat sarcoma viral oncogene homolog | 6zl3 | 7.18 | -8.19 | -7.15 |

**Methods**

***1. Virtual Target identification***

The putative target characterization of retinol was achieved via Pharmacophore-based Virtual screening using PharmMapper [1]. This platform assigns a score to each molecule in the PDB that best fits a pharmacophore model that has been extracted and stored as a library ligand dataset in mol2 format. An almost 77 score matrix is produced by this procedure. After that, when a new molecule is submitted, its fit score is calculated for each pharmacophore, and then each fit score for that pharmacophore is compared to the fit score matrix to determine where it falls on the scale of all the pharmacophore scores. In comparison to chance pharmacophore matching, the pure fit score that results from this procedure carries considerably more weight and assurance. The query structure was submitted to the platform in the pdb format, and the retrieved results were exported as Excel sheet arranging the resulted protein targets according their fit scores.

***2. Docking Study***

The crystal structures of the proteins used for docking were retrieved from PDB (Table S2). Autodock Vina docking machine was used for the docking experiments [2]. The co-crystallized ligand in each protein was used to determine the binding site and the docking grid-box.

The ligand to binding site shape matching root means square (RMSD) threshold was set to 2.0 Å. The interaction energies were determined using the Charmm force field (v.1.02) with 10.0 Å as a non-bonded cutoff distance and distance-dependent dielectric. Then, 5.0 Å was set as an energy grid extending from the binding site [4]. The tested compound retinol was energy minimized inside the selected binding pocket. The editing and visualization of the generated binding poses were performed using Pymol software [3].

***3. Molecular Dynamics Simulation***

NAMD 3.0.0. software was used for performing MDS [4,5]. This software applies the Charmm-36 force field. Protein systems were built using the QwikMD toolkit of the VMD software [5,6], where the protein structure was checked for any missing hydrogens, the protonation states of the amino acid residues were set (pH = 7.4), and the co-crystalized water molecules were removed. Thereafter, the whole structure was embedded in an orthorhombic box of TIP3P water together with 0.15 M Na^+^ and Cl^-^ ions in 20 Å solvent buffer. Afterward, the prepared systems were energy minimized and equilibrated for 5 ns. The parameters and topologies of the ligands were calculated by using the VMD plugin Force Field Toolkit (ffTK). Afterward, the generated parameters and topology files were loaded to VMD to readily read the protein–ligand complexes without errors and then conduct the simulation steps.

***4. Binding Free Energy Calculations***

Molecular Mechanics Poisson-Boltzmann Surface Area (MM-PBSA) embedded in the MMPBSA.py module of AMBER18 was utilized to calculate the binding free energy of the docked complex [7]. 100 frames were processed from the trajectories in total, and the system's net energy was estimated using the following equation:

ΔG_Binding_ = ΔG_Complex_ – ΔG_Receptor_ – ΔG_Inhibitor_

Each of the aforementioned terms requires the calculation of multiple energy components, including van der Waals energy, electrostatic energy, internal energy from molecular mechanics, and polar contribution to solvation energy.

*5. Networks Construction*

All retrieved cancer-relevant proteins (Table S1) were submitted to STRING database [8] in order to find out the potential interactions between them. Cytoscape 3.8.2 (<https://www.cytoscape.org/>) was used for the PPI networks constructions and analysis [9] using the interaction data exported from the STRIN analysis. The lowest interaction score was set to 0.4, and the rest of the parameters were set to the default setting to obtain the PPI networks.

**References**

1. Wang, X.; Shen, Y.; Wang, S.; Li, S.; Zhang, W.; Liu, X.; Lai, L.; Pei, J.; Li, H. PharmMapper 2017 update: A web server for potential drug target identification with a comprehensive target pharmacophore database. Nucleic Acids Res. 2017, 45, W356–W360.

2. Huey, R., Morris, G. M., & Forli, S. (2012). Using AutoDock 4 and AutoDock vina with AutoDockTools: a tutorial. *The Scripps Research Institute Molecular Graphics Laboratory*, *10550*(92037), 1000.‏

3. Yuan, S., Chan, H. S., & Hu, Z. (2017). Using PyMOL as a platform for computational drug design. *Wiley Interdisciplinary Reviews: Computational Molecular Science*, *7*(2), e1298.‏

4. Phillips, J.C.; Braun, R.; Wang, W.; Gumbart, J.; Tajkhorshid, E.; Villa, E.; Chipot, C.; Skeel, R.D.; Kalé, L.; Schulten, K. Scalable molecular dynamics with NAMD. J. Comput. Chem. 2005, 26, 1781–1802.

5. Ribeiro, J. V., Bernardi, R. C., Rudack, T., Schulten, K., & Tajkhorshid, E. (2018). QwikMD-Gateway for Easy Simulation with VMD and NAMD. *Biophysical Journal*, *114*(3), 673a-674a.‏

6. Humphrey, W., Dalke, A., & Schulten, K. (1996). VMD: visual molecular dynamics. *Journal of molecular graphics*, *14*(1), 33-38.

7. Miller III, B.R.; McGee Jr, T.D.; Swails, J.M.; Homeyer, N.; Gohlke, H.; Roitberg, A.E. MMPBSA. py: an efficient program for end-state free energy calculations. *Journal of chemical theory and computation* 2012, *8*, 3314-3321.

8. Mering, C. V., Huynen, M., Jaeggi, D., Schmidt, S., Bork, P., & Snel, B. (2003). STRING: a database of predicted functional associations between proteins. *Nucleic acids research*, *31*(1), 258-261.‏

9. Doncheva, N. T., Morris, J. H., Gorodkin, J., & Jensen, L. J. (2018). Cytoscape StringApp: network analysis and visualization of proteomics data. *Journal of proteome research*, *18*(2), 623-632.‏
